# Neoadjuvant Treatment Versus Upfront Surgery for Resectable Pancreatic Ductal Adenocarcinoma—A Systematic Review and Meta-Analysis of Randomized Controlled Trials

**DOI:** 10.3390/medicina62061049

**Published:** 2026-05-28

**Authors:** Traian Adrian Dușe, Andra Ciocan, Denisa Elena Tiburca, Vlad Dumitru Brata, Radu Vidra, Florin Vasile Zaharie, Andrada Seicean, Ciprian Brisc, Călin Popa, Emil Moiș, Filip Cristian Tocoian, Nadim Al Hajjar

**Affiliations:** 1 Department of Surgery—Surgery III, “Iuliu Hațieganu” University of Medicine and Pharmacy, Croitorilor Street, No. 19–21, 400162 Cluj-Napoca, Romania; duse.traianadrian@elearn.umfcluj.ro (T.A.D.); florinzaharie@elearn.umfcluj.ro (F.V.Z.); cpopa@elearn.umfcluj.ro (C.P.); tocoian_filip_crisitan@elearn.umfcluj.ro (F.C.T.); nadim.alhajjar@umfcluj.ro (N.A.H.); 2 Department of Surgery, “Octavian Fodor” Regional Institute of Gastroenterology and Hepatology, Croitorilor Street, No. 19–21, 400162 Cluj-Napoca, Romania; drmoisemil@elearn.umfcluj.ro; 3 Bihor County Clinical Emergency Hospital, 410167 Oradea, Romania; tiburca.denisaelena@elearn.umfcluj.ro; 4 Department of Gastroenterology, “Octavian Fodor” Regional Institute of Gastroenterology and Hepatology, Croitorilor Street, No. 19–21, 400162 Cluj-Napoca, Romania; brata.vladdumitru@elearn.umfcluj.ro (V.D.B.); andradaseicean@elearn.umfcluj.ro (A.S.); 5 MEDFUTURE Biomedical Research Institute, Department of Personalized Medicine and Rare Diseases, “Iuliu Hațieganu” University of Medicine and Pharmacy, 400347 Cluj-Napoca, Romania; radu.vidra@irgh.ro; 6 Department of Oncology, “Octavian Fodor” Regional Institute of Gastroenterology and Hepatology, Croitorilor Street, No. 19–21, 400162 Cluj-Napoca, Romania; 7 Department of Gastroenterology, “Iuliu Hațieganu” University of Medicine and Pharmacy, Croitorilor Street, No. 19–21, 400162 Cluj-Napoca, Romania; 8 Department of Medical Disciplines, University of Oradea, 410073 Oradea, Romania; briscciprian@uoradea.ro; 9 Department of Surgical Disciplines, “Iuliu Hațieganu” University of Medicine and Pharmacy, Croitorilor Street, No. 19–21, 400162 Cluj-Napoca, Romania

**Keywords:** pancreatic ductal adenocarcinoma, resectable pancreatic cancer, neoadjuvant therapy, perioperative treatment, upfront surgery, systematic review, meta-analysis, randomized controlled trials, overall survival, treatment fidelity

## Abstract

*Background and Objectives:* The role of neoadjuvant or perioperative treatment in anatomically resectable pancreatic ductal adenocarcinoma (PDAC) remains intensely debated, in part because prior evidence syntheses have often pooled resectable and borderline-resectable disease. We aim to evaluate the efficacy and safety of neoadjuvant or perioperative treatment versus upfront surgery in trial-defined resectable PDAC using randomized evidence only. *Materials and Methods:* We performed a systematic review and meta-analysis of parallel-group randomized controlled trials comparing neoadjuvant therapy with upfront surgery in adults with resectable PDAC. PubMed, CENTRAL, Scopus, and Clarivate Web of Science were searched from inception until 30 November 2025. Mixed resectable- and borderline-resectable trials were included only when outcomes for the resectable subgroup were extractable. The primary outcome was overall survival (OS), analyzed on an intention-to-treat basis. Secondary outcomes included time-to-disease-event (TTDE) endpoints, resection rate, R0 resection, pathological node-negative (pN0) status, postoperative morbidity and mortality, and grade ≥3 adverse events. The review protocol was prospectively registered in PROSPERO (CRD420251243805). *Results:* Eight randomized controlled trials enrolling 1395 patients, including 1184 patients with resectable PDAC, met the eligibility criteria. Overall survival was available for six trials (seven comparisons totaling 1066 randomized patients) and was not significantly different between neoadjuvant treatment and upfront surgery (HR 0.80, 95% CI 0.59–1.08). TTDE was likewise not significantly different between strategies (HR 0.80, 95% CI 0.58–1.11). Neoadjuvant treatment reduced the likelihood of proceeding to resection (RR 0.90, 95% CI 0.85–0.95), while R0 resection and pN0 rates were numerically but not significantly higher among the resected patients. Postoperative morbidity and mortality were comparable between groups. Exploratory analyses suggested favorable survival estimates in trials with higher neoadjuvant treatment deliverability and completion. *Conclusions:* In trial-defined resectable PDAC, current randomized evidence does not demonstrate a universal survival advantage for neoadjuvant treatment over upfront surgery. Exploratory trial-level analyses suggested that higher neoadjuvant treatment deliverability and completion were associated with more favorable survival estimates. These findings support a selective, individualized rather than routine use of preoperative strategies in resectable PDAC.

## 1. Introduction

Pancreatic ductal adenocarcinoma (PDAC), also commonly known as pancreatic cancer, contributes substantially to global cancer mortality, with incidence and deaths steadily increasing over recent decades [1,2]. Despite advances in systemic therapy and perioperative care, outcomes remain poor and relapses are common, reflecting aggressive tumor biology and early dissemination, which cannot be captured by conventional staging [3,4].

For anatomically resectable PDAC, management is multimodal. The conventional strategy has long been upfront surgery followed by adjuvant chemotherapy, supported by randomized trials demonstrating improved survival with postoperative multi-agent regimens such as modified FOLFIRINOX or gemcitabine plus capecitabine [5,6,7]. However, the success of a surgery-first approach depends on postoperative recovery and timely initiation and the completion of planned adjuvant treatment. In observational cohorts, a substantial proportion of patients do not receive adjuvant chemotherapy after resection, most commonly due to postoperative complications, delayed functional recovery, or early disease progression, potentially compromising the intended benefits of multimodal therapy [8].

Neoadjuvant or perioperative approaches may offer several advantages, such as earlier treatment of micrometastatic disease, improved deliverability of systemic therapy, higher rates of R0 resection and pathological node-negative status, as well as biological selection by identifying early progression before major surgery [9,10]. Potential disadvantages include toxicity, functional decline, and interval progression that may preclude resection [7,10]. Accordingly, upfront resection with adjuvant therapy remains standard, whereas neoadjuvant therapy is generally reserved for selected high-risk patients, such as markedly elevated CA 19-9, large primary tumor or regional lymph nodes, excessive weight loss, extreme pain, or equivocal imaging findings [5,11].

Randomized trial evidence comparing neoadjuvant or perioperative strategies with upfront surgery has expanded but remains heterogeneous when it comes to systemic regimens, use of radiotherapy, treatment duration, and criteria for resectability. Several trials have enrolled mixed cohorts of resectable and borderline-resectable disease, which introduces stage-mixing bias, as baseline prognosis and the expected benefit of neoadjuvant therapy differ substantially by anatomical stage. Pooling these populations can inflate apparent treatment effects driven predominantly by borderline-resectable subgroups and reduce the applicability of estimates to strictly resectable PDAC [12,13]. Existing meta-analyses have frequently pooled anatomical stages or combined separate neoadjuvant approaches, limiting inference for strictly resectable PDAC and for contemporary treatment strategies, and have not consistently demonstrated an overall survival benefit with neoadjuvant strategies [14,15]. A synthesis restricted to randomized controlled trials in trial-defined resectable PDAC, with attention to intention-to-treat effects and treatment deliverability, is, therefore, warranted.

The aim of our systematic review and meta-analysis is to compare neoadjuvant or perioperative therapy (systemic chemotherapy with or without radiotherapy administered before planned resection, with protocol-defined postoperative therapy, where applicable) versus upfront surgery followed by adjuvant therapy in adults with trial-defined resectable PDAC. The primary outcome was overall survival, assessed on an intention-to-treat basis. Secondary outcomes included time-to-disease-event endpoints (disease-free survival, event-free survival, or progression-free survival), R0 resection rate, pathological node-negative (pN0) status, resection rate among randomized patients, perioperative morbidity and mortality, and grade ≥3 severe adverse events.

## 2. Materials and Methods

The present systematic review and meta-analysis were conducted and reported in accordance with the Cochrane Collaboration Handbook for Systematic Reviews of Interventions [16] and the Preferred Reporting Items for Systematic Reviews and Meta-Analyses (PRISMA) 2020 statement [17]. The review protocol was prospectively registered in the PROSPERO database (CRD420251243805) prior to study selection and data extraction.

### 2.1. Eligibility Criteria

We included parallel-group randomized controlled trials (RCTs) enrolling adult patients (≥18 years) with pancreatic ductal adenocarcinoma (PDAC) considered anatomically resectable according to trial-defined criteria at randomization, with PDAC confirmed histologically, cytologically, or by postoperative pathology. Trials enrolling mixed populations of resectable and borderline-resectable patients were included only if outcomes for the resectable subgroup were available or could be reliably extracted. Subgroup data were considered extractable if hazard ratios (HRs) or event counts for the resectable cohort were explicitly reported, or if Kaplan–Meier curves with corresponding numbers-at-risk were available for the resectable subgroup. Subgroup survival data were not reconstructed when subgroup curves were not clearly separable, when reporting precluded reliable extraction, or when reconstruction would have required unsupported assumptions.

Eligible interventions consisted of protocolized systemic chemotherapy administered for at least one cycle before planned surgical resection, with or without radiotherapy, and with protocol-defined postoperative therapy where applicable. Comparator arms consisted of upfront surgery (UPS) followed by protocol-defined or standard-of-care postoperative therapy.

Trials were excluded if they (i) enrolled exclusively borderline-resectable or locally advanced tumors, or enrolled mixed stages without extractable resectable subgroup data; (ii) lacked published outcome data for any prespecified endpoint; (iii) were non-randomized; (iv) did not include an upfront-surgery comparator arm; or (v) evaluated non-PDAC histologies without separable PDAC results. There were no restrictions on follow-up duration, language, country, or publication date.

### 2.2. Search Strategy and Data Extraction

**Figure 1:**
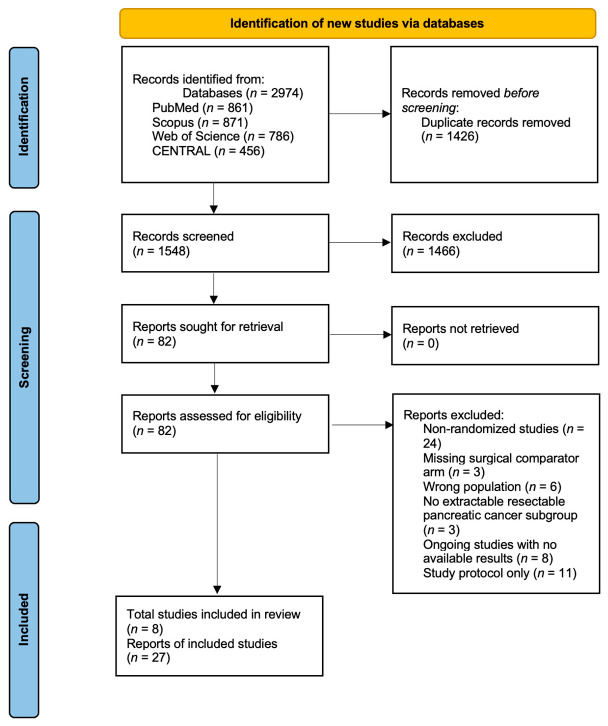
PRISMA flow diagram of the included studies.

We performed a systematic search of PubMed, Cochrane Central Register of Controlled Trials (CENTRAL), Scopus, and Web of Science from database inception until 30 November 2025, using predefined strategies adapted to each database. Additional records were identified through manual review of reference lists from trials and relevant systematic reviews and meta-analyses. Full search strategies are provided in the Supplementary Materials. Two independent reviewers (T.A.D. and D.E.T.) screened titles and abstracts after duplicate removal using Rayyan web-based software 1.0 [18]. Full-text review was subsequently performed to determine eligibility using predefined criteria. Disagreements were resolved by consensus; when necessary, a third reviewer (V.D.B.) provided arbitration. The selection process is summarized in a PRISMA flow diagram (Figure 1).

Two reviewers (T.A.D. and D.E.T.) independently extracted data using a standardized template developed a priori based on the PROSPERO protocol. Extracted items included trial identifiers, sample size, baseline patient and tumor characteristics, resectability definitions, intervention and comparator details (including radiotherapy parameters where applicable), and outcome data. When required data were incomplete or unclear, corresponding authors were contacted for clarification. Discrepancies were resolved by discussion with a third reviewer (V.D.B.).

### 2.3. Outcomes and Subgroup Analysis

The primary outcome was overall survival (OS), defined as time from randomization to death from any cause. Secondary outcomes included trial-defined time-to-disease-event (TTDE) endpoints (disease-free survival [DFS], event-free survival [EFS], or progression-free survival [PFS]); pathological outcomes (margin-negative [R0] resection, pathological node-negative [pN0] status, and histopathological response); perioperative outcomes (resection rate among randomized patients, postoperative morbidity and mortality); and treatment-related grade ≥ 3 adverse events (per The Common Terminology Criteria for Adverse Events (CTCAE), World Health Organization (WHO) or any trial-defined grading).

OS was analyzed as a time-to-event outcome using HRs with 95% confidence intervals (CIs), prioritizing intention-to-treat (ITT) estimates when available. DFS, EFS, and PFS were synthesized under a unified TTDE construct for meta-analytic purposes using each trial’s original endpoint definition, acknowledging definitional differences across trials; endpoint-type subgroup analyses were prespecified to assess potential construct-related heterogeneity.

Resection rate was defined as the proportion of randomized patients undergoing surgical resection. R0 resection and pN0 status were analyzed among resected patients using each trial’s definitions and pathology reporting standards. Histopathological response was extracted as reported. Responses graded according to the College of American Pathologists classification were recorded directly, and prespecified mapping rules were developed for alternative grading systems. Quantitative synthesis, however, was undertaken only when response definitions, thresholds, and analysis populations were considered sufficiently comparable for clinically meaningful pooling.

Perioperative morbidity was preferentially extracted as Clavien–Dindo grade ≥III complications; when this classification was unavailable, trial-reported severe or major complications were pooled if definitions implied invasive intervention, reoperation, organ support, or intensive care admission. Postoperative mortality was extracted as in-hospital death or death within 30 or 90 days of surgery, as reported. Outcome-specific extraction levels are summarized in Supplementary Table S3.

### 2.4. Treatment Deliverability and Completion

Treatment deliverability and completion were extracted as trial-level measures of neoadjuvant therapy feasibility. Deliverability was defined, based on ITT, as the proportion of patients randomized to the experimental arm who completed the planned neoadjuvant protocol, including early attrition prior to treatment initiation; this definition was chosen to reflect real-world feasibility. Completion was defined as the proportion of patients initiating preoperative therapy who completed the planned regimen. When explicit completion data were not reported, values were not imputed. In exploratory analyses, post-neoadjuvant resection among patients initiating NAT was considered as a proxy feasibility measure, recognizing that it does not fully capture treatment completion.

Trials were classified as high versus low fidelity using an a priori threshold of ≥80%, consistent with prior neoadjuvant therapy literature [19,20]. These measures were summarized descriptively and used as prespecified stratification and sensitivity variables to explore their potential role as effect modifiers of survival outcomes; they were not synthesized as pooled comparative endpoints.

Prespecified subgroup analyses explored heterogeneity according to neoadjuvant regimen class (modern multi-agent systemic therapy, legacy systemic therapy, or chemoradiotherapy) and treatment fidelity (deliverability and completion categories). Subgroup analyses based on resectability criteria (modern versus legacy anatomical definitions) were prespecified but not undertaken owing to insufficient data for reliable classification. All subgroup analyses were considered exploratory.

### 2.5. Quality Assessment and Certainty of Evidence

Risk of bias was assessed at the outcome level using the Cochrane Risk of Bias 2 (RoB2) tool [21]. Two reviewers (T.A.D. and V.D.B.) performed assessments independently, with disagreements resolved by consensus. Overall risk of bias was categorized as low risk, some concerns or high risk. Certainty of evidence was evaluated using the Grading of Recommendations Assessment, Development and Evaluation (GRADE) approach [22]. Summary of findings tables are provided in the Supplementary Materials.

### 2.6. Statistical Analysis

Time-to-event outcomes (OS and TTDE) were synthesized using hazard ratios (HRs), and binary outcomes using risk ratios (RRs), each with 95% confidence intervals (CIs). When HRs were not reported, they were estimated from published Kaplan–Meier curves using established reconstruction methods [23,24,25,26]. For efficacy endpoints, intention-to-treat data were preferentially extracted when available. Denominators were prespecified according to endpoint estimate, using the resected population for R0 and pN0 and the randomized population for resection rate.

Meta-analyses were performed using random-effects models with restricted maximum likelihood estimation and Hartung–Knapp adjustment [27]. A two-tailed *p*-value cut-off of <0.05 was considered statistically significant for all analyses. Heterogeneity was assessed using τ^2^ and I^2^ statistics. Multi-arm trials with shared control groups were addressed in accordance with Cochrane recommendations for unit-of-analysis error handling [16]. Prespecified exploratory analyses included subgroup analyses by regimen class and treatment fidelity, random-effects meta-regression for deliverability and completion, influence analyses, and sensitivity analyses. Publication bias for the primary outcome was assessed visually using funnel plots; formal testing was not undertaken due to the limited number of comparisons available [28]. Analyses were conducted in R 4.3.2 using meta, metafor and dmetar packages and cross-checked in RevMan Web [29,30,31]. Additional details regarding the statistical analysis are available in the Supplementary Materials.

## 3. Results

### 3.1. Study Selection and Baseline Characteristics

The systematic search identified 2974 records across all databases. After duplicate removal and title/abstract screening, 83 reports underwent full-text assessment, with eight RCTs meeting all inclusion criteria and included in the final analysis, as presented in Figure 1 [12,13,32,33,34,35,36,37].

Baseline characteristics of included trials are summarized in Table 1. All RCTs were published or available online between 2015 and 2025, with broad geographic representation including trials conducted in Europe (*n* = 6) and Asia (*n* = 2). A total of 1395 patients were enrolled across all trials, of whom 1184 with resectable PDAC were eligible to be included in the quantitative analysis; two trials (Prep-02/JSAP-05 and PREOPANC) enrolled mixed cohorts of resectable and borderline-resectable patients, and only the extractable resectable subgroups were pooled and analyzed. Patients were randomized to neoadjuvant or perioperative treatment (NAT; *n* = 583) or upfront surgery (UPS; *n* = 601).

Experimental strategies were categorized into three prespecified intervention classes: modern multi-agent systemic chemotherapy (*n* = 5 trials), neoadjuvant chemoradiotherapy (*n* = 2), and legacy systemic chemotherapy (*n* = 1). Postoperative treatment was protocolized in both arms across all trials. Median follow-up ranged from 18.7 to 55.4 months. Resectability definitions varied: contemporary trials typically adhered to NCCN or similar criteria, while earlier studies used legacy or protocol-specific definitions focused on major vascular involvement.

Baseline patient demographics are outlined in Table 2. Median age ranged from 60 to 75 years, and most patients had favorable performance status (ECOG 0 or Karnofsky >80%). Tumors were predominantly located in the pancreatic head (50–100% across trials). Clinical lymph node involvement (cN+) at baseline was reported in five trials, ranging from 21.4% to 52.5% in the NAT arms.

The feasibility of delivering neoadjuvant therapy varied across regimens and trial designs (Table 3). Deliverability—defined as the proportion of patients randomized to NAT who completed the planned preoperative regimen—ranged from 48.1% to 89.8%. Among patients initiating NAT, completion rates were consistently high. Surgical resection was achieved in 61–84% of patients in the NAT group and 75–93% in the UPS group. Several trials identified non-PDAC histology following randomization; these cases were handled according to each trial’s prespecified analytic framework (Supplementary Table S1).

### 3.2. Pooled Analysis

#### 3.2.1. Overall Survival

**Figure 2:**
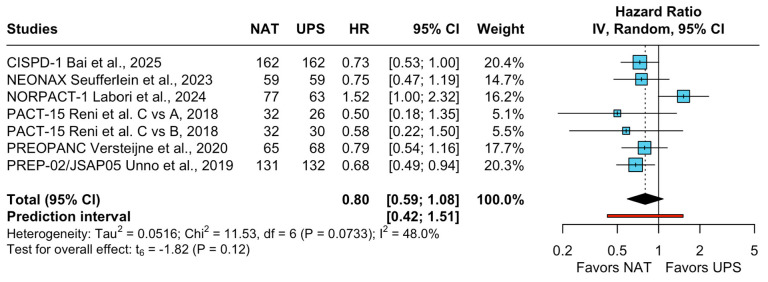
Forest plot of pooled overall survival in neoadjuvant therapy versus upfront surgery in resectable pancreatic ductal adenocarcinoma. NAT—neoadjuvant treatment; UPS—upfront surgery; HR—hazard ratio; CI—confidence interval; PACT-15 Arm A—upfront surgery followed by adjuvant gemcitabine; Arm B—upfront surgery followed by adjuvant PEXG; Arm C—perioperative PEXG. PACT-15 contributes two correlated comparisons from a single multi-arm trial; standard errors were adjusted to account for the shared arm [12,13,32,33,34,35].

Overall survival hazard ratios were available or could be reconstructed for seven comparisons from six trials, comprising 1066 randomized patients with resectable PDAC who contributed to the primary OS meta-analysis (NAT *n* = 526; UPS *n* = 540). All studies measured OS from the time of randomization. Neoadjuvant therapy was not associated with a statistically significant improvement in OS compared with upfront surgery (HR 0.80; 95% CI 0.59–1.08; *p* = 0.12; I^2^ = 48.0%; τ^2^ = 0.052; Figure 2). Point estimates favored NAT in most comparisons; one trial (NORPACT-1) reported a hazard ratio favoring UPS (HR 1.52; 95% CI 1.00–2.32). Sensitivity analysis excluding mixed-population trials that enrolled both resectable and borderline-resectable patients was directionally consistent with the primary OS analysis (Supplementary Figure S10).

#### 3.2.2. Time to Disease Event

**Figure 3:**
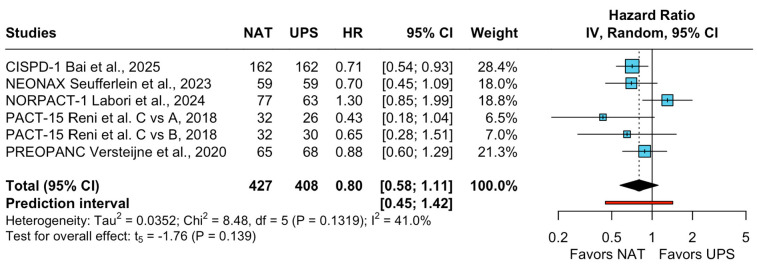
Forest plot of pooled time to disease events in neoadjuvant therapy versus upfront surgery in resectable pancreatic ductal adenocarcinoma. NAT—neoadjuvant treatment; UPS—upfront surgery; HR—hazard ratio; CI—confidence interval; PACT-15 Arm A—upfront surgery followed by adjuvant gemcitabine; Arm B—upfront surgery followed by adjuvant PEXG; Arm C—perioperative PEXG. PACT-15 contributes two correlated comparisons from a single multi-arm trial; standard errors were adjusted to account for the shared arm [12,32,33,34,35].

Six comparisons from five trials (*n* = 803 patients) contributed TTDE hazard ratios; all endpoints were measured from randomization. Neoadjuvant therapy was not associated with a statistically significant difference in TTDE compared with upfront surgery (HR 0.80; 95% CI 0.58–1.11; *p* = 0.14; I^2^ = 41%; τ^2^ = 0.035; Figure 3). Individual estimates were variable, with most comparisons favoring NAT and one favoring UPS (HR 1.30; 95% CI 0.85–1.99). Sensitivity analysis excluding mixed-population trials was also directionally consistent with the primary TTDE analysis (Supplementary Figure S11).

#### 3.2.3. Surgical and Pathological Outcomes

**Figure 4:**
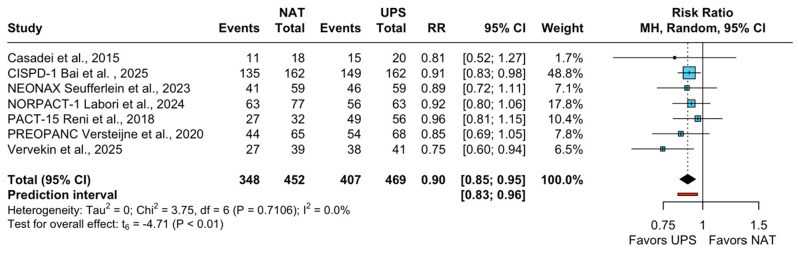
Forest plot of pooled resection rates in neoadjuvant versus upfront surgery in resectable pancreatic ductal adenocarcinoma. NAT—neoadjuvant treatment; UPS—upfront surgery; RR—risk ratio; CI—confidence interval [12,32,33,34,35,36,37].

Resection rates were reported in seven trials (*n* = 921 patients). Neoadjuvant treatment was associated with a significantly lower likelihood of proceeding to resection compared with upfront surgery (77.0% vs. 86.8%; RR 0.90; 95% CI 0.85–0.95; *p* = 0.003; I^2^ = 0%; τ^2^ = 0; Figure 4).

**Figure 5:**
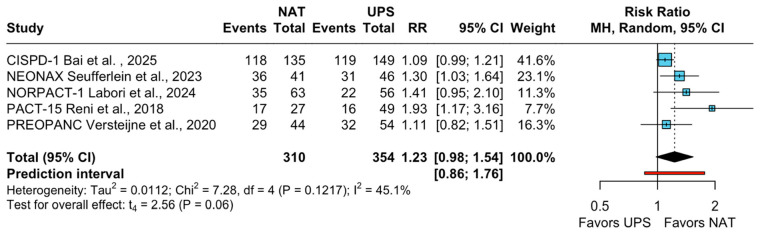
Forest plot of pooled R0 resections in neoadjuvant therapy versus upfront surgery in resectable pancreatic ductal adenocarcinoma. NAT—neoadjuvant treatment; UPS—upfront surgery; RR—risk ratio; CI—confidence interval [12,32,33,34,35] .

R0 resection rates were pooled across five comparisons (*n* = 664 resected patients). Neoadjuvant treatment was associated with a numerically higher probability of achieving margin-negative resection, although the difference did not reach statistical significance (75.8% vs. 62.1%; RR 1.23; 95% CI 0.98–1.54; *p* = 0.06; I^2^ = 45%; τ^2^ = 0.011; Figure 5).

Pathological node-negative (pN0) status was reported in four comparisons. pN0 was observed in 49.8% (134/269) of NAT patients and 42.2% (130/308) of UPS patients. Neoadjuvant treatment was associated with a numerical increase in node-negative resection without reaching statistical significance (RR 1.21; 95% CI 0.80–1.82; *p* = 0.24; I^2^ = 38%; τ^2^ = 0.015; Supplementary Figure S1). Pathological response was not pooled because reporting was highly heterogeneous across trials, including use of different regression grading systems, variable response thresholds, and non-comparable analysis populations. Several studies reported pathological response without resectable-subgroup-specific results. Available data were, therefore, reviewed descriptively but were not considered suitable for quantitative synthesis.

Major postoperative morbidity (Clavien–Dindo ≥ III) was reported in three trials (*n* = 479 patients) and postoperative mortality in four trials (*n* = 566 patients). Major complications occurred in 22.2% of NAT patients versus 18.9% of UPS patients; postoperative mortality was infrequent in both groups (1.9% vs. 2.7%). There were no significant differences in major morbidity (RR 1.19; 95% CI 0.48–2.91; *p* = 0.50; I^2^ = 21%; τ^2^ < 0.001) or postoperative mortality (RR 0.73; 95% CI 0.15–3.45; *p* = 0.56; I^2^ = 0%; τ^2^ = 0) between treatment strategies.

Serious adverse events (grade ≥ 3) did not differ significantly between treatment strategies in random-effects or fixed-effect models. However, only two studies were eligible for the pooled analysis (Supplementary Figure S2).

Visual inspection of the funnel plot for OS did not demonstrate marked asymmetry (Supplementary Figure S3). Given the limited number of contributing comparisons for OS (k = 7), formal testing for small-study effects was not undertaken.

### 3.3. Exploratory Subgroup and Sensitivity Analyses

Given moderate between-study heterogeneity in the primary OS (I^2^ = 48%; τ^2^ = 0.052) and TTDE (I^2^ = 41%; τ^2^ = 0.035) analyses, prespecified exploratory analyses examined potential effect modification by treatment fidelity, regimen class, and endpoint construct. These analyses should be interpreted cautiously given the limited number of contributing trials and the ecological nature of trial-level moderators. Prespecified subgroup analyses by resectability criteria and geography were not undertaken because too few trials could be reliably classified.

#### 3.3.1. Treatment Fidelity and Overall Survival

In trials with high completion rates (≥80%), pooled survival estimates favored neoadjuvant therapy (HR 0.72; 95% CI 0.64–0.80; *p* < 0.001; I^2^ = 0%; τ^2^ = 0), whereas the single trial with low completion (NORPACT-1) favored upfront surgery (HR 1.52; 95% CI 1.00–2.32).

**Figure 6:**
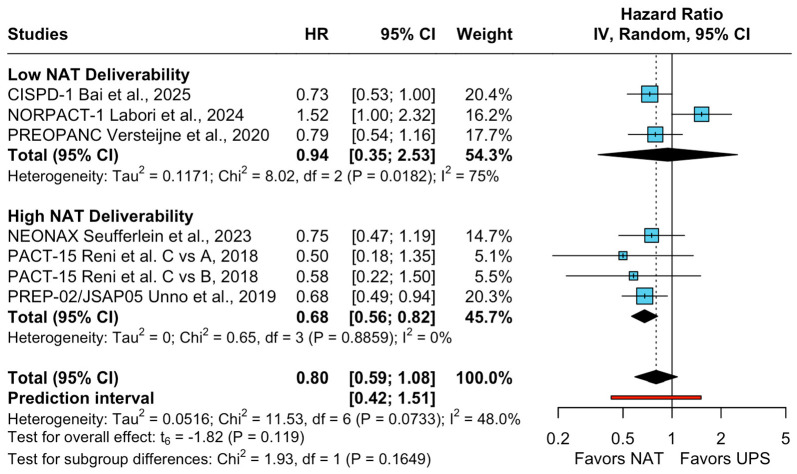
Forest plot of pooled overall survival with subgroup analysis according to neoadjuvant treatment deliverability in resectable pancreatic ductal adenocarcinoma. High versus low deliverability was defined according to the prespecified trial-level threshold. NAT—neoadjuvant treatment; UPS—upfront surgery; HR—hazard ratio; CI—confidence interval; PACT-15 Arm A—upfront surgery followed by adjuvant gemcitabine; Arm B—upfront surgery followed by adjuvant PEXG; Arm C—perioperative PEXG. PACT-15 contributes two correlated comparisons from a single multi-arm trial; standard errors were adjusted to account for the shared arm [12,13,32,33,34,35] .

Stratification by deliverability yielded concordant findings (Figure 6). High-deliverability trials demonstrated a pooled HR favoring NAT (HR 0.68; 95% CI 0.56–0.82; *p* = 0.007; I^2^ = 0%; τ^2^ = 0), whereas low-deliverability trials showed no clear benefit (HR 0.94; 95% CI 0.35–2.53; *p* = 0.16; I^2^ = 75%; τ^2^ = 0.117). The between-subgroup difference did not reach statistical significance (*p* = 0.16).

Subgroup analysis by regimen class demonstrated a significant between-subgroup difference (*p* = 0.02); however, subgroup sizes were unevenly distributed. The modern multi-agent chemotherapy subgroup (k = 4) showed substantial heterogeneity (HR 0.86; 95% CI 0.48–1.54; *p* = 0.60; I^2^ = 70%; τ^2^ = 0.095). The chemoradiotherapy subgroup comprised a single trial (PREOPANC; HR 0.79; 95% CI 0.54–1.16), as did the legacy chemotherapy subgroup (PACT-15, two arms; HR 0.54; 95% CI 0.21–1.39; Supplementary Figure S4).

Continuous exploratory meta-regression showed concordant trial-level associations. Increasing deliverability was significantly associated with lower log-hazard ratios for OS (β = −1.80; SE = 0.39; *p* = 0.006), with complete resolution of residual heterogeneity (τ^2^ = 0). Similarly, completion demonstrated a significant association with survival effect (β = −2.28; SE = 0.30; *p* < 0.001), accounting for all between-study heterogeneity (Supplementary Figures S5 and S6).

Leave-one-out influence analysis indicated that omission of NORPACT-1 shifted the pooled estimate toward statistical significance (HR 0.72; 95% CI 0.64–0.80; *p* < 0.001), whereas exclusion of other individual trials did not materially alter the direction or magnitude of effect (Supplementary Figure S7).

#### 3.3.2. Treatment Fidelity and Time to Disease Event

Meta-regression demonstrated significant associations between treatment fidelity and TTDE. Increasing completion was associated with lower log-hazard ratios (β = −1.87; SE = 0.44; *p* = 0.01), with complete resolution of heterogeneity (τ^2^ = 0; I^2^ = 0%). Deliverability showed a comparable association (β = −1.77; SE = 0.37; *p* = 0.009; residual τ^2^ = 0; I^2^ = 0%).

Leave-one-out analysis demonstrated that omission of NORPACT-1 reduced heterogeneity to zero and resulted in a statistically significant pooled estimate favoring NAT (HR 0.73; 95% CI 0.59–0.90; *p* = 0.01). The pooled estimate including all trials remained non-significant (HR 0.80; 95% CI 0.58–1.11; *p* = 0.14).

Subgroup analysis by TTDE endpoint definition (EFS vs. DFS/PFS) did not demonstrate a significant between-subgroup difference (*p* = 0.12). The EFS subgroup (k = 3) yielded HR 0.68 (95% CI 0.45–1.02; I^2^ = 0%), while the DFS/PFS subgroup (k = 3) yielded HR 0.93 (95% CI 0.43–1.99; I^2^ = 51%).

#### 3.3.3. Surgical Outcomes

For postoperative mortality, restriction to early mortality definitions (30-day or in-hospital; k = 3) yielded a pooled estimate favoring NAT (RR 0.43; 95% CI 0.24–0.77; *p* = 0.025; I^2^ = 0%), although based on only nine events. Fixed-effect modeling attenuated statistical significance (RR 0.42; 95% CI 0.10–1.76; *p* = 0.24), indicating model sensitivity in this sparse dataset.

For resection rates, restriction to high-deliverability trials (k = 2) showed no significant difference (RR 0.93; 95% CI 0.57–1.53; *p* = 0.33). Analysis limited to modern multi-agent regimens (k = 4) demonstrated a modest reduction in resection rates with NAT (RR 0.89; 95% CI 0.81–0.99; *p* = 0.037), although leave-one-out analysis suggested sensitivity to individual trial omission.

For R0 resection, restriction to modern regimens (k = 3) yielded a non-significant effect (RR 1.19; 95% CI 0.87–1.62; *p* = 0.14). Exploratory mixed-denominator analysis (k = 6) resulted in a statistically significant increase favoring NAT (RR 1.24; 95% CI 1.02–1.49; *p* = 0.034), consistent in direction with the primary analysis. This analysis was interpreted as supportive only due to combined denominator structures.

For pN0 status, subgroup analyses were limited by small study numbers (k ≤ 3 in most strata) and did not demonstrate statistically significant differences (all *p* > 0.15).

Across binary outcomes, sensitivity analyses did not materially change the direction of primary findings; however, several analyses were based on small numbers of studies and sparse event counts and should be interpreted cautiously.

### 3.4. Risk of Bias and Certainty of Evidence

Risk of bias was assessed at the outcome level using the RoB2 tool (Supplementary Figures S8 and S9). For OS, two trials were judged at low risk of bias overall; the remaining trials were rated as having some concerns, primarily in domain 2 (deviations from intended interventions) due to the open-label design inherent to surgical oncology trials. All trials were judged at low risk for randomization (D1), missing outcome data (D3), outcome measurement (D4), and selective reporting (D5). No trial was classified as high risk of bias for OS.

For TTDE endpoints, risk-of-bias judgments were less favorable. Three trials (NORPACT-1, NEONAX, and PREOPANC) were rated at high risk of bias overall due to concerns in domain 4 (outcome measurement), reflecting the susceptibility of recurrence-based endpoints to detection bias in open-label settings. The principal source of bias across both endpoints stemmed from the open-label design and potential outcome assessment bias for recurrence endpoints.

According to GRADE methodology, the certainty of evidence for OS was rated as moderate, downgraded for imprecision due to limited trial numbers and wide confidence intervals. Certainty for TTDE was rated as low, downgraded for risk of bias and imprecision. Certainty for binary surgical and pathological outcomes ranged from low to moderate, primarily due to imprecision and small study numbers. A summary of findings table is presented in Supplementary Table S2.

## 4. Discussion

This systematic review and meta-analysis of eight randomized controlled trials comprising 1184 patients with resectable PDAC found that neoadjuvant or perioperative therapy was not associated with a statistically significant improvement in overall survival compared with upfront surgery followed by adjuvant therapy (HR 0.80; 95% CI 0.59–1.08). TTDE outcomes were likewise not significantly different between strategies (HR 0.80; 95% CI 0.58–1.11). Neoadjuvant assignment was associated with a lower probability of proceeding to resection, whereas markers of local oncologic control among resected patients (R0 resection and pN0 status) were numerically higher, and major postoperative morbidity and mortality were comparable.

Point estimates favored neoadjuvant treatment in most comparisons, and exploratory analyses revealed that treatment fidelity, particularly neoadjuvant deliverability measured at the intention-to-treat level, together with treatment completion, was associated with differences in observed survival effects. More favorable estimates were seen in higher-fidelity settings (HR 0.72; 95% CI 0.64–0.80), whereas this pattern was not observed in lower-fidelity settings. These findings suggest that the effectiveness of neoadjuvant strategies in resectable PDAC may be contingent upon successful delivery of the intended preoperative regimen without compromising the likelihood of proceeding to surgical resection.

Our findings are consistent with prior RCT-based syntheses assessing neoadjuvant approaches in pancreatic cancer. Such meta-analyses have generally reported point estimates favoring neoadjuvant strategies without a consistently statistically significant OS advantage across pooled comparisons [14,38,39]. Recent resectable-focused syntheses by Tanadi et al. [40] and Zuo et al. [41] reported higher R0 resection rates without a statistically significant survival advantage, a pattern that is directionally concordant with the present findings. The current analysis prioritized intention-to-treat survival estimates from randomized comparisons, inclusion of mixed-stage trials only where outcomes for the resectable subgroup were separately available and prespecified exploratory assessment of treatment fidelity as a potential contributor to heterogeneity in survival estimates. This approach was intended to facilitate interpretation within strictly resectable pancreatic ductal adenocarcinoma.

One key interpretive issue across the literature is stage mixing. Prior syntheses pooled resectable and borderline-resectable disease or included mixed-stage trials without separable resectable subgroup estimates, which can bias pooled effects toward benefit because borderline-resectable tumors have a greater a priori opportunity for margin conversion and local control following neoadjuvant therapy [15,38,39]. By restricting inclusion to trial-defined resectable cohorts and extracting resectable subgroups from mixed-cohort trials only when outcomes were separable (PREOPANC and Prep-02/JSAP-05), our estimates are more directly applicable to strictly resectable PDAC [12,13].

In the resectable population, pooled OS and TTDE estimates were statistically neutral. Moderate between-study heterogeneity suggests that any strategy-level survival benefit, if present, is likely modest and context-dependent. Confidence intervals remain wide and compatible with clinically meaningful benefit (HR as low as 0.59), no effect or modest harm, reflecting imprecision due to limited trial numbers rather than a definitive null result; accordingly, certainty of evidence for OS was rated as moderate. Additionally, OS is a distal endpoint integrating heterogeneous downstream management, including postoperative systemic therapy exposure and subsequent therapies at recurrence, which may attenuate between-strategy differences even when earlier disease control differs.

Time-to-disease-event endpoints provide complementary information by capturing events occurring during neoadjuvant therapy and prior to surgery. The neutral pooled TTDE estimate, therefore, suggests that earlier disease events were not consistently reduced across available randomized trials in unselected resectable PDAC, while leaving open the possibility that benefit may be restricted to specific contexts, regimens, or implementation conditions. From a clinical perspective, this reinforces the need for improved patient selection within anatomically resectable disease. In that sense, the neutral survival effect observed in this analysis may partly reflect variation in neoadjuvant treatment strategy implementation across trials, rather than differences in systemic therapy efficacy alone. Accordingly, based on currently available randomized evidence restricted to trial-defined resectable PDAC, a universal survival advantage for neoadjuvant or perioperative strategies over upfront surgery cannot be established, and upfront surgery with postoperative systemic therapy remains the standard approach, while neoadjuvant therapy may be considered in selected resectable high-risk patients [5,6,11]. Because postoperative complications and recovery-related decline can reduce the delivery of intended adjuvant chemotherapy after pancreatectomy, earlier systemic therapy remains attractive when preoperative treatment can be reliably delivered [42].

A distinctive contribution of this meta-analysis is the prespecified evaluation of neoadjuvant treatment fidelity as a feasibility construct and its exploration as a potential source of heterogeneity. The association between deliverability (as well as completion) and observed survival effects is exploratory, yet clinically plausible, as neoadjuvant strategies are unlikely to confer benefit unless patients receive sufficient preoperative systemic therapy and still proceed to timely surgery. Incomplete delivery may expose patients to toxicity, nutritional decline, and treatment delays without achieving adequate systemic intensity to influence micrometastatic disease control or resection quality. The NORPACT-1 trial is consistent with this pattern, reporting an OS estimate favoring upfront surgery (HR 1.52; 95% CI 1.00–2.32), together with low completion and deliverability of planned NAT and a lower resection rate in the neoadjuvant arm [33]. By contrast, trials with higher treatment fidelity generally reported more favorable estimates. Prep-02/JSAP-05 achieved high deliverability and completion and demonstrated improved survival in the neoadjuvant arm [13,20], whereas CISPD-1 and PACT-15 also showed directionally similar patterns [32,35]. Exploratory trial-level meta-regression and influence analyses were concordant with this interpretation. Higher deliverability was associated with more favorable overall survival estimates, while in leave-one-out analyses, omission of NORPACT-1 substantially reduced heterogeneity and shifted the pooled estimate toward a more favorable effect for neoadjuvant therapy. These observations also align with the broader sequencing literature indicating that the ability to complete multimodality therapy is an important determinant of outcomes in resectable PDAC [42]. Given the limited number of studies and the ecological nature of trial-level meta-regressions, these findings should be interpreted cautiously, as they may also reflect differences in patient selection, supportive care infrastructure, staging practices, and time to surgery. Influence leave-one-out analysis also showed that the primary OS estimate was sensitive to NORPACT-1. Therefore, the neutral primary OS estimate should be interpreted as the conservative pooled estimate across all eligible randomized evidence, rather than as definitive evidence that neoadjuvant therapy cannot improve survival in selected or high-fidelity settings. However, excluding NORPACT-1 from the primary analysis would introduce post hoc selection bias, because the trial met all eligibility criteria and represents relevant randomized evidence in resectable PDAC. Nevertheless, these analyses suggest that in resectable PDAC, trial results may reflect the performance of perioperative treatment strategies as much as the activity of the systemic regimens themselves, thereby supporting standardized reporting of treatment initiation, completion, dose intensity, and time to surgery in future trials and real-world implementation [43].

Neoadjuvant assignment reduced the likelihood of proceeding to resection at the randomized population level, reflecting attrition during preoperative therapy due to progression, toxicity, functional decline, or patient preference. From an intention-to-treat perspective, some of this attrition may represent appropriate biological selection when early progression identifies aggressive disease unlikely to benefit from surgery. However, it may also represent preventable loss of surgical opportunity when driven by avoidable toxicity, inadequate supportive care, or delays, with pathway optimization and patient selection remaining central when implementing neoadjuvant strategies [43,44]. Therefore, when deliverability is limited, attrition may offset potential benefits of earlier systemic therapy, thereby attenuating strategy-level survival effects in intention-to-treat analyses.

Among patients who underwent resection, neoadjuvant therapy produced directional improvements in pathological measurements (R0 resection and pN0 status). These outcomes, however, were analyzed among resected patients and are, therefore, conditional on reaching surgery. They should be interpreted alongside the lower resection rate observed at the randomized population level. Improved pathological findings among resected patients may reflect treatment effect, biological selection, or both, and do not necessarily indicate a net benefit across all randomized patients. These findings are consistent with the biological rationale proposed for neoadjuvant therapy, particularly its potential to enhance margin-negative resection rates and to address occult regional micrometastatic disease at an earlier stage of treatment. However, these patterns did not achieve statistical significance in the primary analyses, which likely reflects limited statistical power and heterogeneity arising from pathological assessment and reporting across the included trials. Cross-trial comparisons of margin status are further complicated by variability in specimen processing and margin definitions, which can weaken inference and reduce the ability to link R0 improvements to survival in pooled analyses [45]. Perioperative morbidity and mortality were comparable between strategies among patients who reached surgery, consistent with broader evidence suggesting that neoadjuvant approaches do not inherently compromise surgical safety in appropriately selected patients [12,33,34,35,36].

This review has several methodological strengths, including prospective protocol registration, adherence to PRISMA 2020 and Cochrane standards. Pooled analysis of treatment effects included randomized studies exclusively. Eligibility was restricted to anatomically resectable PDAC with extractable survival outcomes, minimizing biological contamination from borderline-resectable disease and allowing interpretation within a clearly defined population. Moreover, time-to-event endpoints were analyzed using definitions anchored to randomization, reducing time-origin bias. Random-effects pooling employed REML estimation with Hartung–Knapp adjustment, providing conservative uncertainty estimates in a sparse evidence base. The prespecified evaluation of treatment deliverability as an implementation-sensitive measure of neoadjuvant feasibility represents an additional methodological strength. Potential unit-of-analysis bias arising from the inclusion of multi-arm trial PACT-15 was handled by retaining the two clinically distinct pairwise contrasts while applying variance adjustment to account for the shared comparator, in accordance with Cochrane guidance. Risk of bias and certainty of evidence were evaluated using RoB2 and GRADE frameworks. Where needed, time-to-event effects were reconstructed from Kaplan–Meier curves using established methods.

Several limitations warrant consideration. First, the number of contributing trials and comparisons was limited, and several prespecified analyses were underpowered or could not be undertaken due to data constraints. Second, clinical heterogeneity in neoadjuvant regimens, resectability definitions, and outcome reporting limited regimen-specific conclusions. Third, most trials were open-label, which may particularly affect recurrence ascertainment and TTDE outcomes; several trials were rated at high risk of bias for TTDE endpoints. Fourth, some time-to-event effects required reconstruction from Kaplan–Meier curves, which may introduce measurement error despite standardized approaches. Fifth, publication bias could not be formally tested given the limited number of comparisons; funnel plot interpretation is necessarily qualitative, and formal tests are underpowered with small numbers of studies. Finally, fidelity analyses, including meta-regressions, were trial-level and exploratory; they cannot establish causal relationships between treatment delivery and survival outcomes and may be confounded by unmeasured trial-level characteristics, including patient selection, staging intensity, supportive care infrastructure, regimen choice, surgical timing, and postoperative treatment.

Future randomized trials in strictly resectable PDAC should prioritize contemporary systemic regimens and standardized perioperative pathways, with transparent reporting of attrition and reasons for non-resection. Standardized reporting of treatment fidelity, including initiation, completion, dose intensity, timing to surgery, and reasons for discontinuation, is essential to interpret strategy-level efficacy and enable reliable synthesis across trials. Trials directly comparing perioperative versus adjuvant-only sequencing with modern multi-agent regimens are underway and may clarify whether optimized delivery shifts outcomes in resectable subpopulations [46,47].

Whether differences between neoadjuvant regimens contribute materially to heterogeneity in resectable PDAC remains uncertain. Perioperative randomized evidence comparing different neoadjuvant regimens suggests that regimen selection alone may not fully explain outcome differences across trials, as illustrated by SWOG S1505, which found no significant difference between perioperative mFOLFIRINOX and gemcitabine/nab-paclitaxel [48]. Individual patient data meta-analysis would enable more precise estimation of treatment effects, exploration of patient-level effect modifiers (age, performance status, CA 19-9, tumor size), and assessment of time-varying hazards [14].

Finally, biomarker-driven selection strategies, including molecular profiling, circulating tumor DNA, and imaging-based response assessment, may identify patients most likely to benefit from neoadjuvant therapy and enable more personalized treatment recommendations [7].

## 5. Conclusions

The current randomized evidence cannot establish a universal survival advantage for neoadjuvant or perioperative treatment in resectable PDAC. Time-to-disease-event outcomes are likewise not significantly different between strategies. Neoadjuvant treatment was associated with lower resection rates at the randomized population level, while R0 resection and pN0 status were numerically higher among patients who underwent surgery. Perioperative morbidity and mortality are comparable between groups. In high-deliverability groups, survival estimates for neoadjuvant strategies are more favorable, whereas this signal was attenuated or reversed in lower-fidelity settings. This suggests that strategy-level effectiveness in resectable PDAC may be sensitive not only to treatment sequencing but also to the feasibility of delivering the intended preoperative regimen without compromising timely resection. However, these findings are hypothesis-generating and do not establish a causal relationship between fidelity and survival. An individualized approach remains appropriate, when neoadjuvant therapy is considered in selected patients, in settings able to optimize treatment delivery and timely transition to surgery.

## Figures and Tables

**Table 1:** Baseline characteristics of the included studies.

Trial (Author, Year)	Region	Phase	Population	Resectability Criteria	Resectable PDAC Randomized, *n*	Intervention Class	NAT Regimen ^d^	Control Regimen ^d^	Median Follow-Up, Months (IQR)
CISPD-1 [32] (Bai, 2025)	China	III	Resectable-only	NCCN	324	Modern ^e^	Gem/Nab-Pac + mFOLFIRINOX (Gem or Gem/Cap)	Surgery (Gem/Cap)	18.7 (13.0–32.0)
NORPACT-1 [33] (Labori, 2024)	Scandinavia	II	Resectable-only	NCCN	140	Modern ^e^	FOLFIRINOX (Gem or Gem/Cap or mFOLFIRINOX)	Surgery (Gem/Cap or mFOLFIRINOX)	22.7 (14.5–33.7)
NEONAX [34] (Seufferlein, 2023)	Germany	II	Resectable-only	German S3 guidelines	118	Modern ^e^	Gem/Nab-Pac (Gem/Nab-Pac)	Surgery (Gem/Nab-Pac)	N/A
PREOPANC-1 [12] (Versteijne, 2020)	Netherlands	III	Mixed (RPC + BRPC)	Dutch Pancreatic Cancer Group guidelines	133 ^a^	CRT	Gem/RT (Gem)	Surgery (Gem)	27/59 (N/A) ^c^
JSAP-05 [13] (Unno, 2019)	Japan	II/III	Mixed (RPC + BRPC)	Trial-defined	263 ^a^	Modern ^e^	Gem/S-1 (S-1)	Surgery (S-1)	38.4 (N/A)
PACT-15 [35] (Reni, 2018)	Italy	II/III	Resectable-only	Trial-defined	88 ^b^	Legacy ^f^	PEXG (PEXG)	Surgery (Gem or PEXG)	55.4 (47.8–69.4)
Casadei, 2015 [36]	Italy	N/A	Resectable-only	Trial-defined—NCCN consistent	38	CRT	Gem/RT (Gem)	Surgery (Gem)	N/A
Vervekin, 2025 [37]	Russia	II	Resectable-only	NCCN	80	Modern ^e^	mFOLFIRINOX (mFOLFIRINOX)	Surgery (mFOLFIRINOX)	N/A

BRPC—borderline resectable pancreatic cancer; Cap—capecitabine; CRT—Chemoradiotherapy; Gem—gemcitabine; IQR—interquartile range; mFOLFIRINOX—modified fluorouracil, leucovorin, irinotecan, and oxaliplatin; N/A—not available; NAT—neoadjuvant treatment; NCCN—National Comprehensive Cancer Network; PEXG—cisplatin, epirubicin, capecitabine, and gemcitabine; RPC—resectable pancreatic cancer; RT—radiotherapy; UPS—upfront surgery. ^a^ Baseline characteristics for mixed resectable/borderline trials are reported at the trial level because subgroup-specific baseline data were not available. ^b^ Data represents pooled upfront surgery and neoadjuvant treatment arms from the PACT-15 study Arm A (*n* = 26), Arm B (*n* = 30) and Arm C (*n* = 32). ^c^ Data represents median follow-up (months) from the original 2020 publication as well as the long-term results publication (2022). ^d^ Data in parentheses indicate protocol-defined postoperative therapy. ^e^ Modern multi-agent systemic chemotherapy. ^f^ Legacy systemic chemotherapy.

**Table 2:** Baseline patient and tumor characteristics in the included studies.

Trial/Author (Year)	Median Age (Range)		Male Sex, *n* (%)		PS Scale	Favorable PS ^f^, *n* (%)		Head Location, *n* (%)		cN+, n (%)	
	NAT	UPS	NAT	UPS		NAT	UPS	NAT	UPS	NAT	UPS
CISPD-1 [32] (2025)	N/A ^a^	N/A	91 (56.2)	102 (63)	ECOG	107 (66)	103 (63.9)	81 (50)	81 (50)	85 (52.5)	79 (48.8)
NORPACT-1 [33] (2024)	68 (60–72)	66 (57–72)	43 (56)	27 (43)	WHO	64 (83)	51 (81)	77 (100)	63 (100)	N/A	N/A
NEONAX [34] (2023)	65 (48–82)	68 (41–88)	34 (57.6)	37 (62.7)	ECOG	46 (78)	46 (78)	41 (69.5)	46 (78)	20 (33.9)	22 (37.3)
PREOPANC-1 [12] (2020)	66 (59–71)	67 (60–73)	64 (54)	74 (58)	WHO	69 (58)	49 (39)	97 (82)	117 (92)	27 (23)	44 (35)
JSAP-05 [13] (2019)	N/A ^a^	N/A	96 (52.7)	95 (53.1)	ECOG	176 (96.7)	169 (94.4)	134 (73.6)	129 (72.1)	39 (21.4)	39 (21.8)
PACT-15 [35] (2018)	64 (39–75)	Arm A: 65 (37–74) Arm B: 68 (49–75) ^b^	25 (78)	27 (48.2) ^c^	Karnofsky	29 (91)	51 (91) ^d^	28 (88)	51 (91) ^e^	N/A	N/A
Casadei (2015) [36]	71.5 (51–78)	67.5 (48–79)	8 (44.4)	14 (70)	N/A	N/A	N/A	15 (83.3)	20 (100)	14 (77.8)	16 (80)
Vervekin (2025) [37]	62 (53–66)	64 (61–72)	16 (41)	18 (43.9)	ECOG	28 (72)	29 (71)	29 (74.4)	32 (78)	17 (43.6)	9 (21.9)

cN+—clinically node-positive; ECOG—Eastern Cooperative Oncology Group; N/A—not available; NAT—neoadjuvant treatment; PS—performance status; UPS—upfront surgery; WHO—World Health Organization. ^a^ Categorical age distributions were not consistently reported across CISPD-1 and JSAP05 trials (age ≤ 65 years and age ≥ 65 years) and therefore could not be summarized quantitatively. ^b^ UPS group for the PACT-15 trial include two comparator arms; values are shown as the range of reported medians and ranges across arms. No pooled summary statistics were calculated. ^c^ Data for male sex represents pooled upfront surgery arms from the PACT-15 study (Arm A, *n* = 14 and Arm B, *n* = 13). ^d^ Data for performance status represents pooled upfront surgery arms from the PACT-15 study (Arm A, *n* = 24 and Arm B, *n* = 27). ^e^ Data for tumor head location represents pooled upfront surgery arms from the PACT-15 study (Arm A, *n* = 25 and Arm B, *n* = 26). ^f^ Favorable PS was defined as either PS 0 according to ECOG or as a Karnofsky score of more than 80%.

**Table 3:** Neoadjuvant treatment feasibility and surgical resection outcomes in the included studies.

Trial (Author, Year)	Randomized NAT, *n*	Randomized UPS, *n*	NAT Started, *n*	NAT Delivered, *n* ^b^	Deliverability, %	Completion, %	NAT Group Resected, *n* (%) ^e^	UPS Group Resected, *n* (%) ^e^
CISPD-1 [32] (Bai, 2025)	162	162	137	127	78.4	92.7	135/162 (83.3)	149/162 (92)
NORPACT-1 [33] (Labori, 2024)	77	63	61	37	48.1	60.7	63/77 (81.8)	56/63 (88.9)
NEONAX [34] (Seufferlein, 2023)	59	59	54	53	89.8	98.1	41/59 (69.5)	46/59 (78)
PREOPANC-1 [12] (2020)	119 ^a^	127 ^a^	91 ^a^	81 ^a^	68.1	89.0	44/65 (67.7)	54/68 (79.4)
JSAP-05 [13] (Unno, 2019)	182 ^a^	179 ^a^	172 ^a^	161 ^a,d^	88.5	93.6	N/A ^f^	N/A ^f^
PACT-15 [35] (Reni, 2018)	32	56 ^c^	29	28 ^d^	87.5	96.6	27/32 (84.4)	49/56 (87.5)
Casadei, 2015 [36]	18	20	18	14	77.8	77.8	11/18 (61.1)	15/20 (75)
Vervekin, 2025 [37]	39	41	39	27 ^d^	69.2	69.2	27/39 (69.2)	38/41 (92.7)

BRPC—borderline resectable pancreatic cancer; N/A—not available; NAT—neoadjuvant treatment; RPC—resectable pancreatic cancer; UPS—upfront surgery. Resection rates are reported for the resectable subgroup when available; feasibility metrics are reported at the trial level (mixed ITT) when subgroup-specific feasibility data were not reported. ^a^ Including patients with resectable- and borderline-resectable pancreatic cancer; data unavailable for the resectable subgroup, entire NAT group included in the analysis. ^b^ Delivered planned neoadjuvant therapy (deliverability) was defined as the proportion of patients randomized to the neoadjuvant arm who successfully completed the protocol-specified neoadjuvant treatment. When not explicitly reported, deliverability was conservatively estimated from CONSORT flow diagrams as the number of patients proceeding to surgical exploration following neoadjuvant therapy. ^c^ Total number of patients randomized to UPS in the PACT-15 trial include the two immediate surgery arms (Arm A and Arm B). ^d^ Neoadjuvant completion not explicitly reported; data estimated from number of patients who underwent surgical resection following neoadjuvant therapy. ^e^ Reported as percentage of randomized resectable pancreatic cancer (RPC) patients undergoing resection, where available. ^f^ Resection flow for the resectable subgroup was not extractable for JSAP-05; therefore, subgroup resection rates were not presented, and the trial was not included in pooled resection-rate analyses.

## Data Availability

The original contributions presented in this study are included in the article. Further data can be required from the corresponding author.

## Supplementary Materials

The following supporting information can be downloaded at: https://www.mdpi.com/article/10.3390/medicina62061049/s1, Figure S1: Forest plot of pooled pathological node-negative (pN0) status among resected patients in neoadjuvant/perioperative therapy vs. upfront surgery in resectable pancreatic ductal adenocarcinoma; Figure S2: Forest plot of pooled grade ≥3 adverse events in neoadjuvant/perioperative therapy vs. upfront surgery in resectable pancreatic ductal adenocarcinoma; Figure S3: Funnel plot of overall survival in randomized trials comparing neoadjuvant/perioperative therapy vs. upfront surgery in resectable pancreatic ductal adenocarcinoma; Figure S4: Forest plot of pooled overall survival with subgroup analysis according to neoadjuvant regimen class in resectable pancreatic ductal adenocarcinoma; Figure S5: Meta-regression bubble plot of overall survival according to neoadjuvant treatment deliverability in randomized trials of resectable pancreatic ductal adenocarcinoma; Figure S6: Meta-regression plot of overall survival according to neoadjuvant treatment completion in randomized trials of resectable pancreatic ductal adenocarcinoma; Figure S7: Leave-one-out influence analysis of pooled overall survival in randomized trials comparing neoadjuvant/perioperative therapy vs. upfront surgery in resectable pancreatic ductal adenocarcinoma; Figure S8: Risk-of-bias assessment for overall survival using the Cochrane Risk of Bias 2 (RoB2) tool in randomized trials of resectable pancreatic ductal adenocarcinoma; Figure S9: Risk-of-bias assessment for time-to-disease-event outcomes using the Cochrane Risk of Bias 2 (RoB2) tool in randomized trials of resectable pancreatic ductal adenocarcinoma; Figure S10: Forest plot of pooled overall survival after excluding mixed-population trials in neoadjuvant therapy versus upfront surgery in resectable pancreatic ductal adenocarcinoma; Figure S11: Forest plot of pooled time-to-disease-event outcomes after excluding mixed-population trials in neoadjuvant therapy versus upfront surgery in resectable pancreatic ductal adenocarcinoma; Table S1: Reporting of Non-PDAC Cases Across Included Studies; Table S2: GRADE Summary of Findings for Neoadjuvant or Perioperative Treatment Versus Upfront Surgery in Resectable Pancreatic Ductal Adenocarcinoma; Table S3: Outcome-level data source and analysis population across included trials; Table S4: Origin of hazard-ratio estimates for overall survival and time-to-disease-event endpoints; Table S5: Studies and related records excluded because resectable pancreatic cancer subgroup data were not separately extractable or because no eligible outcome data were available. PRISMA 2020 Checklist.

## Abbreviations

The following abbreviations are used in this manuscript:

ASCO	American Society of Clinical Oncology
BRPC	Borderline Resectable Pancreatic Cancer
CA 19-9	Carbohydrate Antigen 19-9
Cap	Capecitabine
CENTRAL	Cochrane Central Register of Controlled Trials
CI	Confidence Interval
cN+	Clinically Node-Positive
CRT	Chemoradiotherapy
CT	Computed Tomography
CTCAE	Common Terminology Criteria for Adverse Events
D1	Bias Arising from The Randomization Process
D2	Bias Due To Deviations from Intended Interventions
D3	Bias Due To Missing Outcome Data
D4	Bias In Measurement of the Outcome
D5	Bias In Selection of the Reported Result
DFS	Disease-Free Survival
ECOG	Eastern Cooperative Oncology Group
EFS	Event-Free Survival
ESMO	European Society for Medical Oncology
Gem	Gemcitabine
GRADE	Grading Of Recommendations Assessment, Development and Evaluation
HKSJ	Hartung–Knapp–Sidik–Jonkman
HR	Hazard Ratio
IPD	Individual Patient Data
IQR	Interquartile Range
ITT	Intention-To-Treat
mFOLFIRINOX	Modified Fluorouracil, Leucovorin, Irinotecan, And Oxaliplatin
N/A	Not Available
NAT	Neoadjuvant Treatment
NCCN	National Comprehensive Cancer Network
OS	Overall Survival
PDAC	Pancreatic Ductal Adenocarcinoma
PEXG	Cisplatin, Epirubicin, Capecitabine, And Gemcitabine
PFS	Progression-Free Survival
pN0	Pathological Node-Negative
PRISMA	Preferred Reporting Items for Systematic Reviews and Meta-Analyses
PROSPERO	International Prospective Register of Systematic Reviews
PS	Performance Status
R0	Margin-Negative Resection
RCT	Randomized Controlled Trial
REML	Restricted Maximum Likelihood
RevMan	Review Manager
RFS	Relapse-Free Survival
RoB2	Risk Of Bias 2
RR	Risk Ratio
RPC	Resectable Pancreatic Cancer
RT	Radiotherapy
TTDE	Time-To-Disease-Event
UPS	Upfront Surgery
WHO	World Health Organization
