# Supplementary material for: Neoadjuvant Treatment Versus Upfront Surgery for Resectable Pancreatic Ductal Adenocarcinoma—A Systematic Review and Meta-Analysis of Randomized Controlled Trials
Source: Medicina (Kaunas). 2026 May 28;62(6):1049. doi: 10.3390/medicina62061049 (PMC13304359; doi:10.3390/medicina62061049)
Supplement: Supplementary file 1 [file medicina-62-01049-s001.zip › medicina-4282921-supplementary.pdf]

medicina
MDPI

Review

Neoadjuvant treatment versus upfront surgery for resectable pancreatic ductal adenocarcinoma – a systematic review and meta-analysis of randomized controlled trials

Traian Adrian Dușe[1,2], Andra Ciocan[1,2,*], Denisa Elena Țiburca[3], Vlad Dumitru Brata[4], Radu Vidra[5,6], Florin Vasile Zaharie[1,2], Andrada Seicean[4,7], Ciprian Brisc[8], Călin Popa[1,2], Emil Moiș[2,9], Filip Cristian Tocoian[1,2] and Nadim Al Hajjar[1,2]

A. Supplementary Tables

Supplementary Table S1. Report upon non-PDAC cases across the included studies

Trial (Author, Year)	Population	Non-PDAC cases – NAT, n	Non-PDAC cases – UPS, n	Where handled
CISPD-1 (Bai, 2025)	Resectable only	4	3	Final pathology after surgery
NORPACT-1 (Labori, 2024)	Resectable only	4	7	Per-protocol exclusions
NEONAX (Seufferlein, 2023)	Resectable only	N/A	N/A	Post-randomization exclusions
PREOPANC-1 (Versteijne, 2020)	Mixed (RPC+BRPC)	4	10	Final pathology after resection
JSAP-05 (Unno, 2019)	Mixed (RPC+BRPC)	0	2	Post-randomization ineligibility
PACT-15 (Reni, 2018)	Resectable only	N/A	N/A	Not reported
Casadei (2015)	Resectable only	N/A	N/A	Not reported
Vervekin et al. (2025)	Resectable only	N/A	N/A	Not reported

BRPC – borderline resectable pancreatic cancer; *N/A* – not available; *NAT* – neoadjuvant treatment; *PDAC* – pancreatic ductal adenocarcinoma; *RPC* – resectable pancreatic cancer; *UPS* – upfront surgery. Non-PDAC cases are reported as described by each trial and reflect heterogeneous handling (post-randomization exclusion, per-protocol exclusion, or final pathology after resection); counts are presented descriptively and were not pooled or used to modify effect estimates.

Supplementary Table S2. GRADE summary of findings for neoadjuvant treatment versus upfront surgery in resectable pancreatic ductal adenocarcinoma

Outcome	Control risk	Intervention risk	Relative effect (95% CI)	Participants (studies)	Certainty
Overall survival	N/A	N/A	HR 0.80 (0.59 to 1.08)	1,066 (6 trials; 7 comparisons)	Moderate[a] ⊕⊕⊕◯
TTDE (DFS/EFS/PFS)	N/A	N/A	HR 0.80 (0.58 to 1.11)	803 (5 trials; 6 comparisons)	Low[b] ⊕⊕◯◯
Resection rate	868 per 1,000	770 per 1,000	RR 0.90 (0.85 to 0.95)	921 (7 trials)	Moderate[c] ⊕⊕⊕◯
R0 resection	621 per 1,000	758 per 1,000	RR 1.23 (0.98 to 1.54)	664 (5 comparisons)	Low[d] ⊕⊕◯◯
pN0 status	422 per 1,000	498 per 1,000	RR 1.21 (0.80 to 1.82)	577 (4 comparisons)	Low[e] ⊕⊕◯◯
Major postoperative morbidity	189 per 1,000	222 per 1,000	RR 1.19 (0.48 to 2.91)	479 (3 trials)	Low[f] ⊕⊕◯◯
Postoperative mortality	27 per 1,000	19 per 1,000	RR 0.73 (0.15 to 3.45)	566 (4 trials)	Low[g] ⊕⊕◯◯

CI – confidence interval; *DFS* – disease-free survival; *EFS* – event-free survival; *GRADE* – Grading of Recommendations Assessment, Development and Evaluation; *HR* – hazard ratio; *N/A* – not available; *NAT* – neoadjuvant treatment; *pN0* – pathological node-negative; *RR* – risk ratio; *TTDE* – time-to-disease-event; *UPS* – upfront surgery. Absolute effects are reported only for binary outcomes; absolute effects for time-to-event outcomes were not estimated because pooled hazard ratios were not anchored to a common follow-up time point or baseline survival risk. [a.] Downgraded for imprecision: the confidence interval crosses no effect and includes both clinically important benefit and no clear effect. Absolute effects for overall survival were not estimated because pooled hazard ratios were not anchored to a common follow-up time point or baseline survival risk. [b.] Downgraded for risk of bias and imprecision. Three trials were judged at high risk of bias for TTDE because recurrence-based endpoints are susceptible to detection bias in open-label trials. Absolute effects were not estimated because pooled hazard ratios were not anchored to a common follow-up time point or baseline survival risk. [c.] Downgraded for indirectness because resection rate reflects both treatment effect and preoperative attrition during neoadjuvant therapy. [d.] Downgraded for inconsistency and imprecision because pathological assessment and margin definitions varied across trials, and the confidence interval crosses no effect. [e.] Downgraded for inconsistency and imprecision because few studies contributed and the confidence interval was wide. [f.] Downgraded for very serious imprecision because few studies and sparse events produced a very wide confidence interval. [g.] Downgraded for very serious imprecision because sparse events produced an extremely wide confidence interval.

Supplementary Table S3. Outcome-level data source and analysis population across included trials

Trial	Population enrolled	OS	TTDE	Resection rate	R0	pN0	Major morbidity	Postoperative Mortality	Grade ≥3 adverse events	NAT deliverability / completion
CISPD-1 (Bai, 2025)	Resectable-only	Full resectable-only trial population	Full resectable-only trial population	Full resectable-only trial population	Resected subgroup	Resected subgroup	Resected subgroup	Resected subgroup	Trial safety population	Full resectable-only trial population
NORPACT-1 (Labori, 2024)	Resectable-only	Full resectable-only trial population	Full resectable-only trial population	Full resectable-only trial population	Resected subgroup	Resected subgroup	Resected subgroup	Resected subgroup	Trial safety population	Full resectable-only trial population
NEONAX (Seufferlein, 2023)	Resectable-only	Full resectable-only trial population	Full resectable-only trial population	Full resectable-only trial population	Resected subgroup	N/A	Reported, excluded[d]	Resected subgroup	N/A	Full resectable-only trial population
PREOPANC-1 (Versteijne, 2020)	Mixed RPC + BRPC	Extractable resectable subgroup	Extractable resectable subgroup	Extractable resectable subgroup	Resected subgroup	Resected subgroup	Reported, excluded[d]	N/A	Reported, excluded[d]	Mixed RPC/BRPC trial-level population[a]
JSAP-05 (Unno, 2019)	Mixed RPC + BRPC	Extractable resectable subgroup	N/A	N/A	N/A	N/A	N/A	N/A	N/A	Mixed RPC/BRPC trial-level population[a]
PACT-15 (Reni, 2018)	Resectable-only	Full resectable-only trial population[c]	Full resectable-only trial population[c]	Full resectable-only trial population; UPS arms	Resected subgroup	Resected subgroup	Resected subgroup	Resected subgroup	Reported, excluded[d]	Full resectable-only trial population[b]

				pooled as shown in Table 3						
Casadei (2015)	Resectable-only	N/A	Full resectable-only trial population	Full resectable-only trial population	Resected subgroup	N/A	Reported, excluded[d]	Resected subgroup	Reported, excluded[d]	Full resectable-only trial population
Vervekin et al. (2025)	Resectable-only	N/A	Full resectable-only trial population	Full resectable-only trial population	Reported, excluded[d]	Reported, excluded[d]	Reported, excluded[d]	Reported, excluded[d]	N/A	Full resectable-only trial population[b]

BRPC – borderline resectable pancreatic cancer; *N/A* – not available; *NAT* – neoadjuvant treatment; *OS* – overall survival; *pN0* – pathological node-negative; *R0* – margin-negative resection; *RPC* – resectable pancreatic cancer; *TTDE* – time-to-disease-event; *UPS* – upfront surgery. [a] Mixed trial-level data from both resectable and borderline-resectable patients; subgroup-specific deliverability/completion data for resectable patients were not reported. [b] Neoadjuvant treatment delivery/completion was estimated from the number of patients proceeding to resection when explicit completion data were not reported. [c] Multi-arm trial; shared comparator handled using variance adjustment according to pre-specified methods. [d] Data were reported in the trial manuscript but excluded from analysis because definitions were insufficiently specified, incompletely reported, or clinically inconsistent with the prespecified outcome definitions.

Supplementary Table S4. Origin of hazard-ratio estimates for overall survival and time-to-disease-event endpoints

**Trial**	**OS HR origin**	**TTDE HR origin**
CISPD-1 (Bai, 2025)	Trial reported HR	Directly reported HR
NORPACT-1 (Labori, 2024)	Trial reported HR	Directly reported HR
NEONAX (Seufferlein, 2023)	Reconstructed from Kaplan–Meier curve[b]	Reconstructed from Kaplan–Meier curve
PREOPANC-1 (Versteijne, 2020)	Trial reported HR for resectable subgroup[c]	Directly reported or extractable resectable-subgroup HR
JSAP-05 (Unno, 2019)	Trial reported HR for resectable subgroup[c]	N/A
PACT-15 (Reni, 2018)	Reconstructed from Kaplan–Meier curve[b]	Reconstructed from Kaplan–Meier curve
Casadei (2015)	N/A	Directly reported HR or extractable time-to-event estimate

Vervekin et al. (2025)	N/A	Directly reported HR or extractable time-to-event estimate

HR – hazard ratio; *KM* – Kaplan–Meier; *N/A* – not available or not included in the pooled analysis; *OS* – overall survival; *TTDE* – time-to-disease-event. [a] HR and corresponding confidence interval or variance were available from the trial report and were used without Kaplan–Meier reconstruction. [b] HR and variance were estimated from digitized Kaplan–Meier curves using the prespecified reconstruction approach. [c] For mixed resectable/borderline-resectable trials, only extractable resectable-subgroup estimates were used.

Supplementary Table S5. Studies and related records excluded because resectable pancreatic cancer subgroup data were not separately extractable or because no eligible outcome data were available.

Study / record	Main report	DOI	Publication type	Reason for exclusion
NEPAFOX	Goetze et al., 2024	10.1245/s10434-024-15011-7	RCT	Mixed resectable/borderline-resectable population; resectable-PDAC subgroup outcomes not separately extractable
Golcher / AIO	Golcher et al., 2015	10.1007/s00066-014-0737-7	RCT	Locally resectable/potentially resectable population; strictly resectable-PDAC subgroup outcomes not separately extractable
Kumar et al.	Kumar et al., 2024	10.4103/jcrt.jcrt_1824_23	RCT	Mixed resectable/borderline-resectable population; resectable-PDAC subgroup outcomes not separately extractable

B. Search strategy

PUBMED

((pancreatic cancer OR pancreatic adenocarcinoma OR pancreatic carcinoma) OR ("adenocarcinoma" AND pancreas) OR ("pancreas"[tiab] AND carcinoma[tiab]))AND (resectable[tiab] OR "resectable pancreatic") AND (neoadjuvant OR preoperative OR perioperative OR neoadjuvant[tiab] OR preoperative[tiab] OR perioperative[tiab]) AND (chemotherapy OR chemoradiation OR chemoradiotherapy OR radiochemotherapy OR FOLFIRINOX OR mFOLFIRINOX OR "modified FOLFIRINOX" OR gemcitabine OR "gemcitabine/cisplatin" OR "nab-paclitaxel" OR "S-1") AND (((surgery OR resection) AND (upfront OR immediate OR first OR alone OR primary)) OR "surgery alone" OR "upfront surgery" OR "primary surgery" OR "immediate surgery")

SCOPUS

TITLE-ABS-KEY (("pancreatic cancer" OR "pancreatic adenocarcinoma" OR "pancreatic carcinoma" OR (adenocarcinoma AND pancreas) OR (pancreas AND carcinoma)) AND

(resectable OR "resectable pancreatic") AND (neoadjuvant OR preoperative OR perioperative) AND (chemotherapy OR chemoradiation OR chemoradiotherapy OR radiochemotherapy OR FOLFIRINOX OR mFOLFIRINOX OR "modified FOLFIRINOX" OR gemcitabine OR "gemcitabine/cisplatin" OR "nab-paclitaxel" OR "S-1") AND (((surgery OR resection) AND (upfront OR immediate OR first OR alone OR primary)) OR "surgery alone" OR "upfront surgery" OR "primary surgery" OR "immediate surgery"))

CLARIVATE WEB OF SCIENCE

TS=(("pancreatic cancer" OR "pancreatic adenocarcinoma" OR "pancreatic carcinoma" OR (adenocarcinoma AND pancreas) OR (pancreas AND carcinoma)) AND (resectable OR "resectable pancreatic") AND (neoadjuvant OR preoperative OR perioperative) AND (chemotherapy OR chemoradiation OR chemoradiotherapy OR radiochemotherapy OR FOLFIRINOX OR mFOLFIRINOX OR "modified FOLFIRINOX" OR gemcitabine OR "gemcitabine/cisplatin" OR "nab-paclitaxel" OR "S-1") AND (((surgery OR resection) AND (upfront OR immediate OR first OR alone OR primary)) OR "surgery alone" OR "upfront surgery" OR "primary surgery" OR "immediate surgery"))

COCHRANE CENTRAL

("pancreatic cancer" OR "pancreatic adenocarcinoma" OR "pancreatic carcinoma" OR (adenocarcinoma AND pancreas) OR (pancreas AND carcinoma)) AND (resectable OR "resectable pancreatic") AND (neoadjuvant OR preoperative OR perioperative) AND (chemotherapy OR chemoradiation OR chemoradiotherapy OR radiochemotherapy OR FOLFIRINOX OR mFOLFIRINOX OR "modified FOLFIRINOX" OR gemcitabine OR "gemcitabine/cisplatin" OR "nab-paclitaxel" OR "S-1") AND (((surgery OR resection) AND (upfront OR immediate OR first OR alone OR primary)) OR "surgery alone" OR "upfront surgery" OR "primary surgery" OR "immediate surgery")

C. Statistical analysis

Time-to-event outcomes (OS and TTDE) were synthesized using HRs; binary outcomes (resection rate, R0 resection, pN0 status, grade ≥3 adverse events, postoperative morbidity, and mortality) were synthesized using risk ratios (RRs). All estimates are reported with 95% CIs. When HRs were not explicitly reported, data were extracted from published Kaplan–Meier curves using established digitization methods [1]. Curves were independently digitized by two reviewers using PlotDigitizer web-based software [2], with axis calibration performed prior to extraction. Approximate individual patient data were reconstructed using IPDfromKM software [3], according to the method described by Guyot et al. and used to estimate HRs with corresponding variance [4]. Reconstructed estimates were cross-checked for concordance when HRs were also reported; discrepancies were resolved by consensus. For efficacy endpoints, ITT data were preferentially extracted when available.

For pathological and surgical outcomes, denominators were prespecified according to the estimates of each endpoint: resected population for R0 and pN0, randomized population for resection rate. Mixed-denominator sensitivity analyses were performed when required by reporting constraints.

All meta-analyses were performed using random-effects models with restricted maximum likelihood (REML) estimation for between-study variance. Hartung–Knapp–Sidik–Jonkman (HKSJ) adjustment was applied to variance estimates to minimize the risk of false-positive findings [5]. Heterogeneity was evaluated using τ^2 and I^2 statistics; Cochran's Q test was reported for completeness. Outcomes with few contributing comparisons were accompanied by sensitivity analyses using fixed-effects models and interpreted with caution.

In multi-arm trials with shared comparator groups, unit-of-analysis issues were addressed in accordance with Cochrane Handbook recommendations [6]. When clinically distinct neoadjuvant arms were compared separately against a shared control, standard errors were inflated by $\sqrt{2}$ prior to inverse-variance pooling to avoid double-counting and preserve appropriate weighting.

Prespecified investigations of heterogeneity included subgroup analyses by regimen class and treatment fidelity categories. Deliverability and completion were additionally examined as continuous moderators using random-effects meta-regression and interpreted as exploratory. Influence analyses were performed using leave-one-out analyses and Baujat plots. Sensitivity analyses additionally examined early postoperative mortality windows (30-day or in-hospital), restriction to modern systemic regimens, and exclusion of lower-fidelity trials.

Publication bias was assessed by visual inspection of funnel plots for the primary outcome. Given the limited number of contributing comparisons ($k<10$), formal testing for small-study effects was not undertaken [7].
All statistical analyses were performed using R version 4.3.2 (R Foundation for Statistical Computing, Vienna, Austria) within the RStudio environment (Posit Software, Boston, MA, USA), primarily using the meta, metafor, and dmetar packages [8-10]. Forest plots and key analyses were cross-checked using Review Manager (RevMan) Web version 9.17.0 (Cochrane Collaboration, Copenhagen, Denmark).

B. Supplementary Figures

Study	NAT Events	NAT Total	UPS Events	UPS Total	RR	95% CI	Weight
CISPD–1 Bai et al. , 2025	74	135	77	149	1.06	[0.85; 1.32]	47.6%
NORPACT–1 Labori et al., 2024	18	63	8	56	2.00	[0.94; 4.24]	7.9%
PACT–15 Reni et al., 2018	13	27	13	49	1.81	[0.99; 3.34]	11.6%
PREOPANC Versteijne et al., 2020	29	44	32	54	1.11	[0.82; 1.51]	32.9%
**Total (95% CI)**		**269**		**308**	**1.21**	**[0.80; 1.82]**	**100.0%**
**Prediction interval**						**[0.71; 2.04]**	

Heterogeneity: $Tau^2 = 0.0146$; $Chi^2 = 4.82$, df = 3 (P = 0.1858); $I^2 = 37.7\%$
Test for overall effect: $t_3 = 1.45$ (P = 0.24)

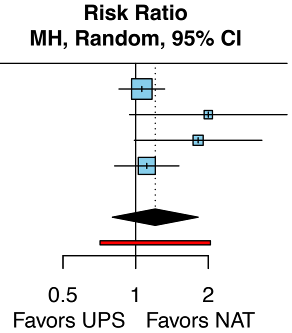

NAT – neoadjuvant treatment; *UPS* – upfront surgery; *pN0* – pathological node-negative; *RR* – risk ratio; *CI* – confidence interval

Supplementary Figure S1. Forest plot of pooled pathological node-negative (pN0) status among resected patients in neoadjuvant therapy versus upfront surgery in resectable pancreatic ductal adenocarcinoma

Study	AE (NAT)	Total (NAT)	AE (UPS)	Total (UPS)	RR	95% CI	Weight
CISPD–1 Bai et al. , 2025	68	143	35	116	1.58	[1.14; 2.18]	60.0%
NORPACT–1 Labori et al., 2024	42	73	19	47	1.42	[0.95; 2.12]	40.0%
**Total (95% CI)**	**110**	**216**	**54**	**163**	**1.51**	**[0.80; 2.85]**	**100.0%**
**Prediction interval**						**[0.29; 7.77]**	

Heterogeneity: $Tau^2 = 0$; $Chi^2 = 0.15$, df = 1 (P = 0.6981); $I^2 = 0.0\%$
Test for overall effect: $t_1 = 8.29$ (P = 0.08)

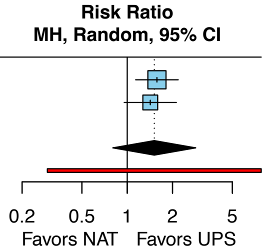

NAT – neoadjuvant treatment; *UPS* – upfront surgery; *RR* – risk ratio; *CI* – confidence interval

Supplementary Figure S2. Forest plot of pooled grade ≥3 adverse events in neoadjuvant versus upfront surgery in resectable pancreatic ductal adenocarcinoma

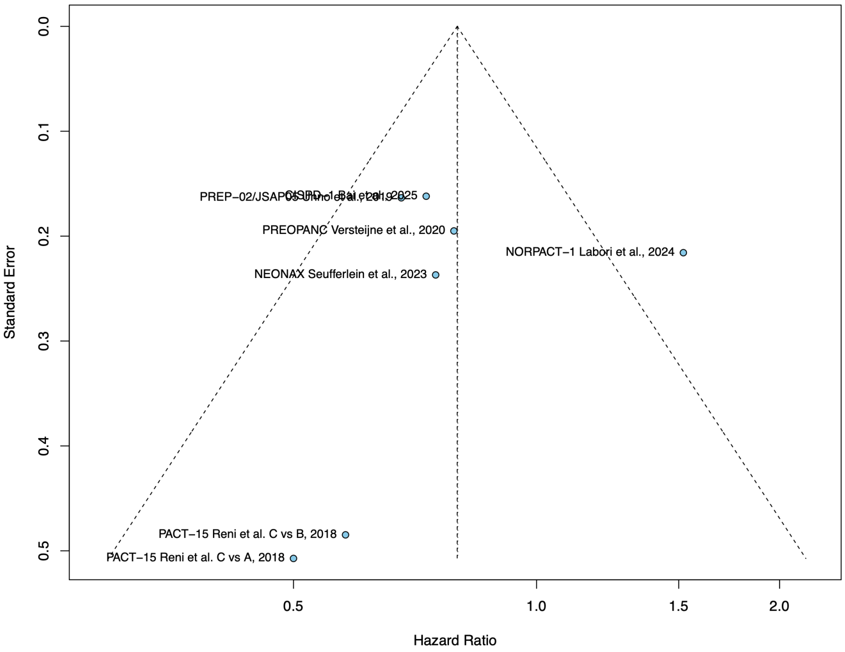

HR – hazard ratio. PACT-15 Arm A – upfront surgery followed by adjuvant gemcitabine; Arm B – upfront surgery followed by adjuvant PEXG; Arm C – perioperative PEXG. PACT-15 Arm A – upfront surgery followed by adjuvant gemcitabine; Arm B – upfront surgery followed by adjuvant PEXG; Arm C – perioperative PEXG.

Supplementary Figure S3. Funnel plot of overall survival in randomized trials comparing neoadjuvant therapy versus upfront surgery in resectable pancreatic ductal adenocarcinoma

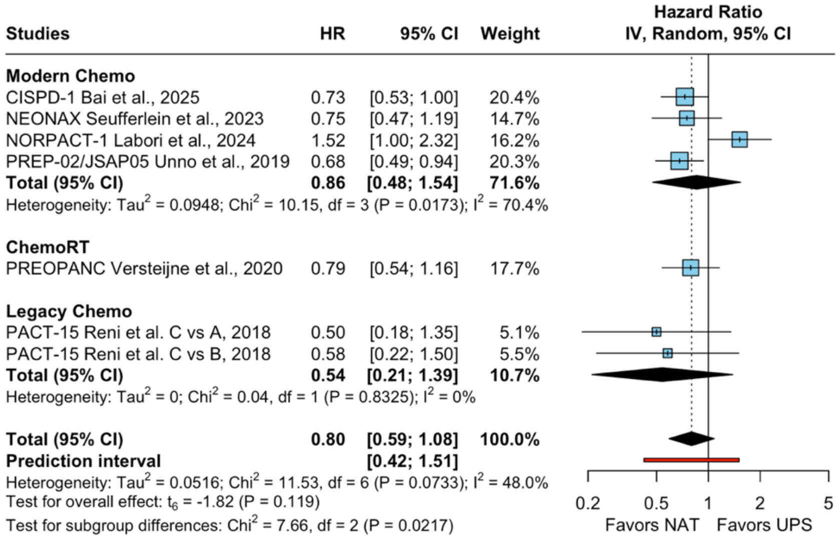

NAT – neoadjuvant treatment; *UPS* – upfront surgery; *HR* – hazard ratio; *CI* – confidence interval; *ChemoRT* – chemoradiotherapy. PACT-15 Arm A – upfront surgery followed by adjuvant gemcitabine; Arm B – upfront surgery followed by adjuvant PEXG; Arm C – perioperative PEXG. PACT-15 Arm A – upfront surgery followed by adjuvant gemcitabine; Arm B – upfront surgery followed by adjuvant PEXG; Arm C – perioperative PEXG.

Supplementary Figure S4. Forest plot of pooled overall survival with subgroup analysis according to neoadjuvant regimen class in resectable pancreatic ductal adenocarcinoma

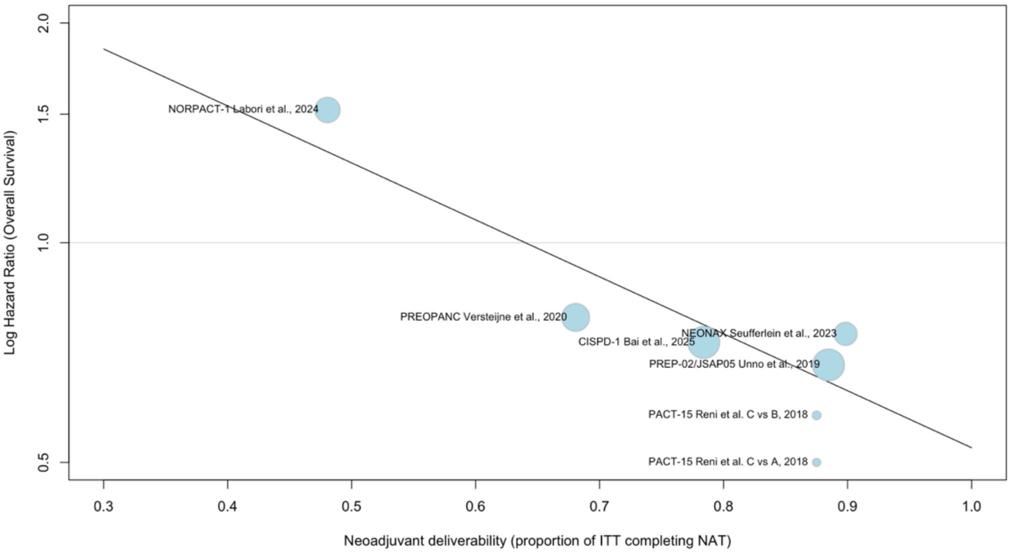

NAT – neoadjuvant treatment; *HR* – hazard ratio; *ITT* – intention-to-treat. PACT-15 Arm A – upfront surgery followed by adjuvant gemcitabine; Arm B – upfront surgery followed by adjuvant PEXG; Arm C – perioperative PEXG. PACT-15 Arm A – upfront surgery followed by adjuvant gemcitabine; Arm B – upfront surgery followed by adjuvant PEXG; Arm C – perioperative PEXG.

Supplementary Figure S5. Meta-regression bubble plot of overall survival according to neoadjuvant treatment deliverability in randomized trials of resectable pancreatic ductal adenocarcinoma

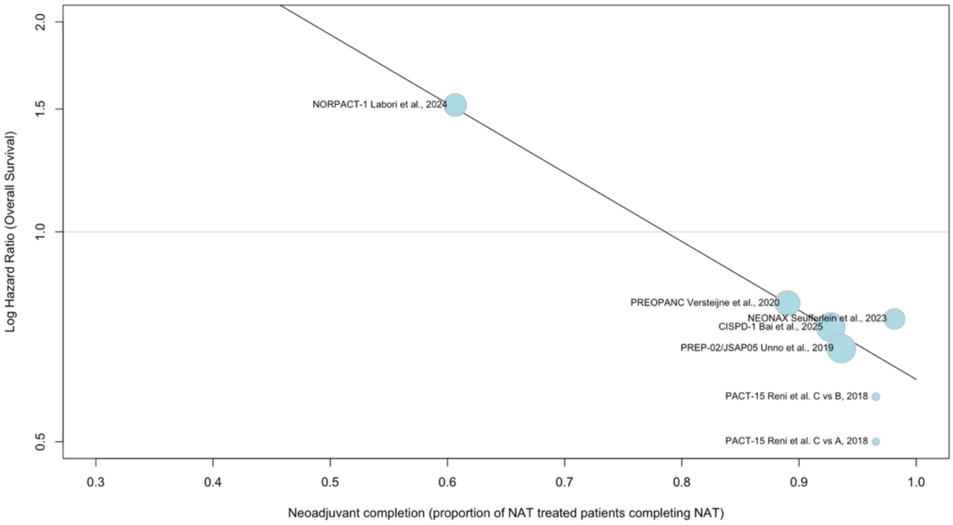

NAT – neoadjuvant treatment; *HR* – hazard ratio. PACT-15 Arm A – upfront surgery followed by adjuvant gemcitabine; Arm B – upfront surgery followed by adjuvant PEXG; Arm C – perioperative PEXG. PACT-15 Arm A – upfront surgery followed by adjuvant gemcitabine; Arm B – upfront surgery followed by adjuvant PEXG; Arm C – perioperative PEXG.

Supplementary Figure S6. Meta-regression plot of overall survival according to neoadjuvant treatment completion in randomized trials of resectable pancreatic ductal adenocarcinoma

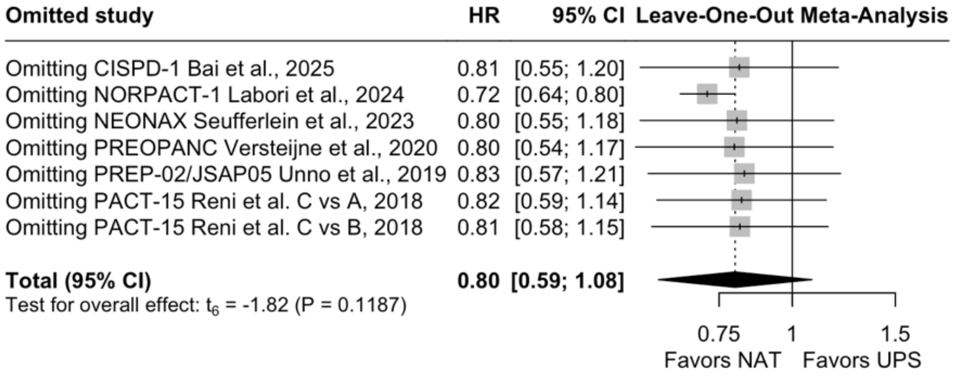

NAT – neoadjuvant treatment; *UPS* – upfront surgery; *HR* – hazard ratio; *CI* – confidence interval. PACT-15 Arm A – upfront surgery followed by adjuvant gemcitabine; Arm B – upfront surgery followed by adjuvant PEXG; Arm C – perioperative PEXG. PACT-15 Arm A – upfront surgery followed by adjuvant gemcitabine; Arm B – upfront surgery followed by adjuvant PEXG; Arm C – perioperative PEXG.

Supplementary Figure S7. Leave-one-out influence analysis of pooled overall survival in randomized trials comparing neoadjuvant therapy versus upfront surgery in resectable pancreatic ductal adenocarcinoma

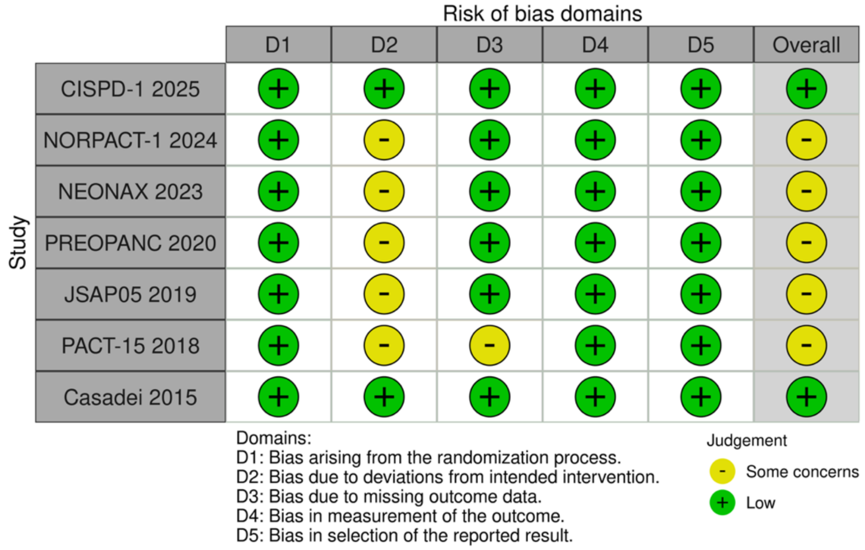

D1 – bias arising from the randomization process; D2 – bias due to deviations from intended intervention; D3 – bias due to missing outcome data; D4 – bias in measurement of the outcome; D5 – bias in selection of the reported result

Supplementary Figure S8. Risk-of-bias assessment for overall survival using the Cochrane Risk of Bias 2 (RoB2) tool in randomized trials of resectable pancreatic ductal adenocarcinoma

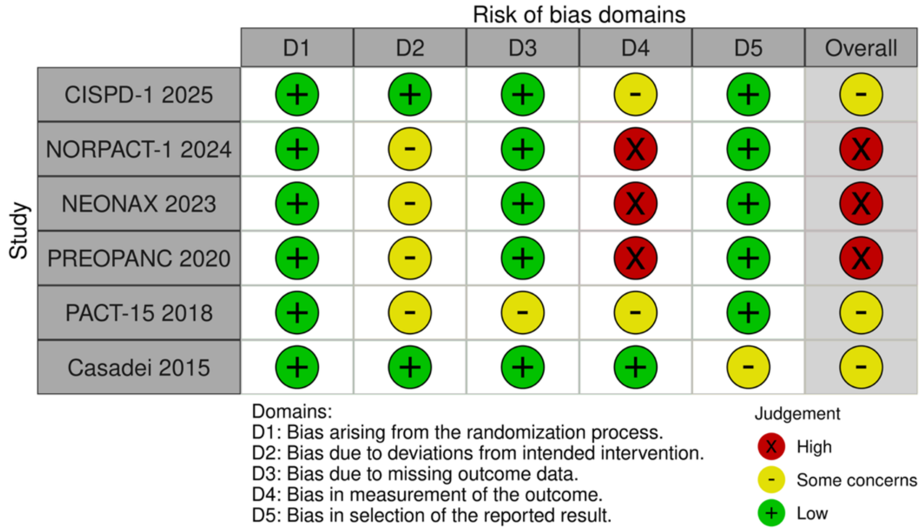

D1 – bias arising from the randomization process; D2 – bias due to deviations from intended intervention; D3 – bias due to missing outcome data; D4 – bias in measurement of the outcome; D5 – bias in selection of the reported result

Supplementary Figure S9. Risk-of-bias assessment for time-to-disease-event outcomes using the Cochrane Risk of Bias 2 (RoB2) tool in randomized trials of resectable pancreatic ductal adenocarcinoma

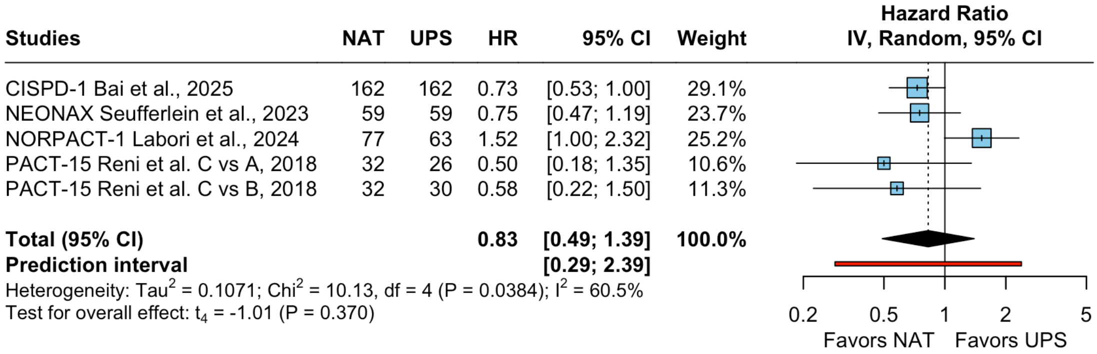

NAT – neoadjuvant treatment; *UPS* – upfront surgery; *HR* – hazard ratio; *CI* – confidence interval; PACT-15 Arm A – upfront surgery followed by adjuvant gemcitabine; Arm B – upfront surgery followed by adjuvant PEXG**;** Arm C – perioperative PEXG. Mixed-population trials were defined as trials enrolling both resectable and borderline-resectable pancreatic cancer, with resectable PDAC available only as a subgroup; PREOPANC and Prep-02/JSAP-05 were excluded.

Supplementary Figure S10. Forest plot of pooled overall survival after excluding mixed-population trials in neoadjuvant therapy versus upfront surgery in resectable pancreatic ductal adenocarcinoma.

NAT – neoadjuvant treatment; *UPS* – upfront surgery; *HR* – hazard ratio; *CI* – confidence interval

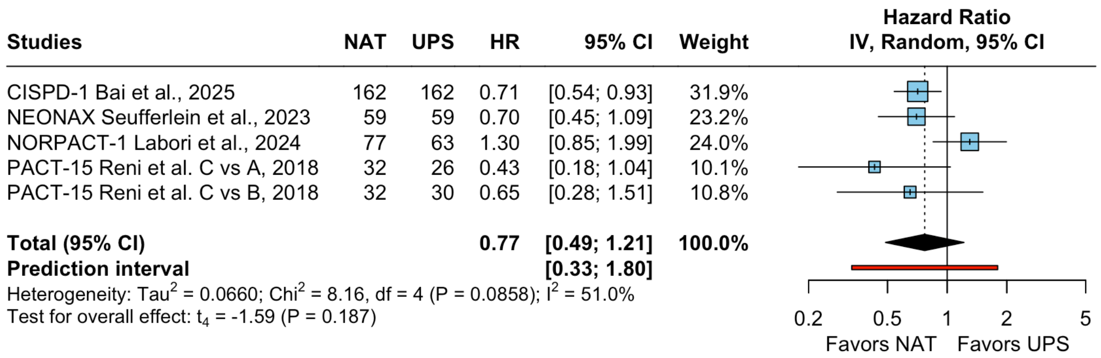

PACT-15 Arm A – upfront surgery followed by adjuvant gemcitabine; Arm B – upfront surgery followed by adjuvant PEXG; Arm C – perioperative PEXG.. Mixed-population trials were defined as trials enrolling both resectable and borderline-resectable pancreatic cancer, with resectable PDAC available only as a subgroup; PREOPANC was excluded from this analysis because Prep-02/JSAP-05 did not contribute to the TTDE meta-analysis.

Supplementary Figure S11. Forest plot of pooled time-to-disease-event outcomes after excluding mixed-population trials in neoadjuvant therapy versus upfront surgery in resectable pancreatic ductal adenocarcinoma.

Supplementary Materials: The following supporting information can be downloaded at: https://www.mdpi.com/article/doi/s1, Figure S1: Forest plot of pooled pathological node-negative (pN0) status among resected patients in neoadjuvant/perioperative therapy vs. upfront surgery in resectable pancreatic ductal adenocarcinoma; Figure S2: Forest plot of pooled grade ≥3 adverse events in neoadjuvant/perioperative therapy vs. upfront surgery in resectable pancreatic ductal adenocarcinoma; Figure S3: Funnel plot of overall survival in randomized trials comparing neoadjuvant/perioperative therapy vs. upfront surgery in resectable pancreatic ductal adenocarcinoma; Figure S4: Forest plot of pooled overall survival with subgroup analysis according to neoadjuvant regimen class in resectable pancreatic ductal adenocarcinoma; Figure S5: Meta-regression bubble plot of overall survival according to neoadjuvant treatment deliverability in randomized trials of resectable pancreatic ductal adenocarcinoma; Figure S6: Meta-regression plot of overall survival according to neoadjuvant treatment completion in randomized trials of resectable pancreatic ductal adenocarcinoma; Figure S7: Leave-one-out influence analysis of pooled overall survival in randomized trials comparing neoadjuvant/perioperative therapy vs. upfront surgery in resectable pancreatic ductal adenocarcinoma; Figure S8: Risk-of-bias assessment for overall survival using the Cochrane Risk of Bias 2 (RoB2) tool in randomized trials of resectable pancreatic ductal adenocarcinoma; Figure S9: Risk-of-bias assessment for time-to-disease-event outcomes using the Cochrane Risk of Bias 2 (RoB2) tool in randomized trials of resectable pancreatic ductal adenocarcinoma; Figure S10: Forest plot of pooled overall survival after excluding mixed-population trials in neoadjuvant therapy versus upfront surgery in resectable pancreatic ductal adenocarcinoma; Figure S11: Forest plot of pooled time-to-disease-event outcomes after excluding mixed-population trials in neoadjuvant therapy versus upfront surgery in resectable pancreatic ductal adenocarcinoma; Table S1: Reporting of Non-PDAC Cases Across Included Studies; Table S2: GRADE Summary of Findings for Neoadjuvant or Perioperative Treatment Versus Upfront Surgery in Resectable Pancreatic Ductal Adenocarcinoma; Table S3: Outcome-level data source and analysis population across included trials; Table

S4: Origin of hazard-ratio estimates for overall survival and time-to-disease-event endpoints; Table S5: Studies and related records excluded because resectable pancreatic cancer subgroup data were not separately extractable or because no eligible outcome data were available.

Data Availability Statement: The original contributions presented in this study are included in the article and Supplementary Materials. Further data can be required from the corresponding author.

References

1. Tierney, J.F.; Stewart, L.A.; Ghersi, D.; Burdett, S.; Sydes, M.R. Practical methods for incorporating summary time-to-event data into meta-analysis. *Trials* **2007**, *8*, 16. https://doi.org/10.1186/1745-6215-8-16
2. PlotDigitizer. Version 3.1.6. Available online: https://plotdigitizer.com (accessed on 12 January 2026).
3. IPDfromKM. Version 1.2.4.0. Available online: https://biostatistics.mdanderson.org/shinyapps/IPDfromKM(accessed on 12 January 2026).
4. Guyot, P.; Ades, A.E.; Ouwens, M.J.; Welton, N.J. Enhanced secondary analysis of survival data: Reconstructing the data from published Kaplan-Meier survival curves. *BMC Med. Res. Methodol.* **2012**, *12*, 9. https://doi.org/10.1186/1471-2288-12-9
5. IntHout, J.; Ioannidis, J.P.; Borm, G.F. The Hartung-Knapp-Sidik-Jonkman method for random effects meta-analysis is straightforward and considerably outperforms the standard DerSimonian-Laird method. *BMC Med. Res. Methodol.* **2014**, *14*, 25. https://doi.org/10.1186/1471-2288-14-25
6. Higgins, J.P.T.; Thomas, J.; Chandler, J.; et al., Eds. *Cochrane Handbook for Systematic Reviews of Interventions*, version 6.4 (updated August 2023); Cochrane, 2023. Available online: www.training.cochrane.org/handbook(accessed on 27 December 2025).
7. Sterne, J.A.; Sutton, A.J.; Ioannidis, J.P.; Terrin, N.; Jones, D.R.; Lau, J.; Carpenter, J.; Rücker, G.; Harbord, R.M.; Schmid, C.H.; et al. Recommendations for examining and interpreting funnel plot asymmetry in meta-analyses of randomised controlled trials. *BMJ* **2011**, *343*, d4002. https://doi.org/10.1136/bmj.d4002
8. Balduzzi, S.; Rücker, G.; Schwarzer, G. How to perform a meta-analysis with R: A practical tutorial. *Evid. Based Ment. Health* **2019**, *22*, 153–160. https://doi.org/10.1136/ebmental-2019-300117
9. Viechtbauer, W. Conducting Meta-Analyses in R with the metafor Package. *J. Stat. Softw.* **2010**, *36*, 1–48. https://doi.org/10.18637/jss.v036.i03
10. Harrer, M.; Cuijpers, P.; Furukawa, T.; Ebert, D. *Doing Meta-Analysis with R: A Hands-On Guide*, 1st ed.; Chapman and Hall/CRC: Boca Raton, FL, USA, 2021. https://doi.org/10.1201/9781003107347

Disclaimer/Publisher's Note: The statements, opinions and data contained in all publications are solely those of the individual author(s) and contributor(s) and not of MDPI and/or the editor(s). MDPI and/or the editor(s) disclaim responsibility for any injury to people or property resulting from any ideas, methods, instructions or products referred to in the content.
